# Two-stage diffuse fluorescence tomography for monitoring of drug distribution in photodynamic therapy of tumors

**DOI:** 10.1117/1.JBO.30.1.015003

**Published:** 2025-01-30

**Authors:** Stefan Šušnjar, Muhammad Daniyal Ghauri, Björn Thomasson, Sanathana Konugolu Venkata Sekar, Stefan Andersson-Engels, Johannes Swartling, Nina Reistad

**Affiliations:** a Lund University, Department of Physics, Lund, Sweden; b SpectraCure AB, Lund, Sweden; c Tyndall National Institute, Biophotonics@Tyndall, Cork, Ireland; d BioPixS Ltd – Biophotonics Standards, IPIC, Cork, Ireland; e University College Cork, School of Physics, Cork, Ireland

**Keywords:** diffuse optical tomography, fluorescence, photodynamic therapy, photosensitizing drug, inverse problem, tissue phantoms

## Abstract

**Significance:**

The spatial distribution of the photosensitizing drug concentration is an important parameter for predicting the photodynamic therapy (PDT) outcome. Current diffuse fluorescence tomography methods lack accuracy in quantifying drug concentration. The development of accurate methods for monitoring the temporal evolution of the drug distribution in tissue can advance the real-time light dosimetry in PDT of tumors, leading to better treatment outcomes.

**Aim:**

We develop diffuse optical tomography methods based on interstitial fluorescence measurements to accurately reconstruct the spatial distribution of fluorescent photosensitizing drugs in real-time.

**Approach:**

A two-stage reconstruction algorithm is proposed. The capabilities and limitations of this method are studied in various simulated scenarios. For the first time, experimental validation is conducted using the clinical system for interstitial PDT of prostate cancer on prostate tissue-mimicking phantoms with the photosensitizer verteporfin.

**Results:**

The average relative error of the reconstructed fluorophore absorption was less than 10%, whereas the fluorescent inclusion reconstructed volume relative error was less than 35%.

**Conclusions:**

The proposed method can be used to monitor the temporal evolution of the photosensitizing drug concentration in tumor tissue during photodynamic therapy. This is an important step forward in the development of the next generation of real-time light dosimetry algorithms for photodynamic therapy.

## 1 Introduction

Photodynamic therapy (PDT) is a promising approach in cancer treatment.^1^ It relies on photodynamic action, a dynamic interaction between light, a photosensitizing agent, and oxygen, resulting in tissue destruction.^2^ None of these three components is individually toxic, but together they initiate a photochemical reaction whose product is a highly reactive singlet oxygen. Its significant toxicity leads to direct tumor cell death via apoptosis or necrosis, damage to tumor vasculature, and activation of innate and adaptive responses against tumors.^3^^,^^4^ PDT’s minimal invasiveness and selectivity in killing malignant cells while sparing surrounding tissue make it effective in treating early-stage tumors and a good choice for inoperable tumors.^3^ PDT has many assets that make it suitable for cancer treatment, either as a first option or combined with other methods (e.g., chemotherapy, radiotherapy, surgery). The light is non-ionizing, and no cumulative toxicity allows repeated treatments of recurrent malignancies.^3^^,^^5^ The short penetration of this light in tissue has led to the evolution of PDT in two directions: one is the development of photosensitizers (PSs) activated at wavelengths where light is less attenuated,^6^ and the other is the interstitial placement of optical fibers for treating solid tumors deeply embedded into the body.^7^^–^^9^ It is essential to have access to light dosimetry algorithms to get a favorable response to PDT.^10^

The destruction of malignant tissue in PDT depends on the type and dose of PSs used, the time between their administration and light delivery, total light dose and its fluence rate, and tumor oxygen concentration.^3^ Therefore, to improve the light dosimetry during PDT treatments, information on PS distribution, light fluence rate, and oxygen concentration should be available in real-time.^11^^–^^15^ Some PSs have the important property to accumulate to a higher degree in tumors, rather than in healthy tissue.^6^ Most PSs are fluorescent in nature.^7^ These properties of PS enable better tumor localization for dose planning, as well as assessment of treatment progression.

Real-time dosimetry during interstitial PDT treatments has already been employed in clinical applications.^16^^–^^19^ In prostate cancer treatment, Johansson et al.^18^ have shown that real-time dosimetry allows for the delivery of a specific dose of light to the target tissue while sparing the organs at risk. However, the distribution of the PS drug within the patient tissue should have been considered during the light dose planning, and the lack of reliable methods for its estimation is the reason why it still has not been considered. To solve this, the present work aims to develop methods for real-time monitoring of the PS spatial distribution during the PDT treatment. This could be beneficial in the future to improve PDT dosimetry algorithms considering additional factors, such as the fluorescent properties of PS.^20^ PS distribution and concentration in the tumor are a major determinant of photochemical oxygen depletion, and as such, knowledge of it is beneficial for accurately predicting the treatment outcome.^21^

In this work, we develop diffuse optical tomography (DOT) models and methods for the real-time reconstruction of the spatial distribution of the PS drug by utilizing its fluorescent properties, which we will refer to as diffuse fluorescence tomography (DFT). The reconstruction algorithm we propose here consists of two stages. The first stage (S1) relies on standard tomographic reconstruction methods^22^^–^^24^ without structural a priori information. The second stage (S2), presented here for the first time, uses the results from S1 as inputs and significantly reduces the discrepancy between the reconstructed fluorophore absorption and its ground-truth (GT), compared to S1. The reconstruction methods are implemented in computer software, then tested and quantitatively evaluated in numerical simulations, and their limitations and potential improvements are discussed. Prostate tissue-mimicking phantoms with verteporfin, a PS used in PDT (excited by light around 690 nm),^25^ were prepared following the work by Ghauri et al.,^26^ to provide realistic experimental validation of the developed methods. The measurements are performed with SpectraCure’s P18 system for interstitial PDT of prostate cancer.^8^^,^^16^ This is, to the best of our knowledge, the first time a quantitative tomographic reconstruction of the concentration of the PS verteporfin has been demonstrated on a clinical system for interstitial PDT of prostate cancer.

## 2 Model

### 2.1 Diffusion Equation for Fluorescence

We assume a highly scattering medium, with the absorption coefficient $\mu_{a}$, the reduced scattering coefficient $\mu_{s}^{\prime }$, satisfying $\mu_{a}\ll \mu_{s}^{\prime }$, and all the conditions of the diffusion approximation.^27^ The diffusion coefficient is defined by $D=\frac{1}{3\mu_{s}^{\prime }}$ and the effective attenuation coefficient $\mu_{\mathrm{eff}}=\sqrt{\frac{\mu_{a}}{D}}$. We consider not necessarily a homogeneous medium, so the aforementioned coefficients will be in general dependent on the spatial coordinates: $\mu_{a}(\overset{\to }{r})$, $\mu_{s}^{\prime }(\vec{r})$, and $D(\vec{r})$. We distinguish the optical properties and quantities at excitation from those at fluorescence emission wavelengths, by adding a letter in the subscript—x for the excitation and m for the fluorescence emission. If the fluorophore absorption coefficient $\mu_{af}$ is defined as the probability of absorption of a photon at the excitation wavelength, per unit pathlength it has covered, and if the fraction of such absorbed photons which result in the fluorescent re-emission is γ, known as the fluorescence quantum yield,^28^ then the fluorescent yield η can be defined as the product of the two,^22^

$$\eta (\overset{\to }{r})=\gamma \mu_{af}(\overset{\to }{r}),$$ (1)

by assuming γ is independent of the fluorophore spatial distribution. Considering a continuous-wave (CW) light source $q_{0}(\vec{r})$, the steady-state diffusion equation for the fluence rate at excitation wavelength $\Phi_{x}$ is^27^

$$(-\nabla D_{x}(\vec{r})\nabla +\mu_{ax}(\vec{r}))\Phi_{x}(\vec{r})=q_{0}(\vec{r}),$$ (2)

Where we assumed $\mu_{af}\ll \mu_{ax}$, i.e., the equation is unaffected by the presence of fluorophores. The source of fluorescence emission $q_{m}(\vec{r})$ is defined where the fluorophores exist in space, and its strength is proportional to the excitation fluence and the fluorescent yield,

$$q_{m}(\vec{r})=\eta (\vec{r})\Phi_{x}(\vec{r}).$$ (3)

The fluence rate at fluorescence emission wavelength $\Phi_{m}$ is found from the following diffusion equation ^7^^,^^29^

$$(-\nabla D_{m}(\overset{\to }{r})\nabla +\mu_{\mathrm{am}}(\overset{\to }{r}))\Phi_{m}(\overset{\to }{r})=\eta (\overset{\to }{r})\Phi_{x}(\overset{\to }{r}).$$ (4)

Assuming $\mu_{\mathrm{af}}\ll \mu_{\mathrm{am}}$, the emission light fluence from Eq. (4) can be obtained by

$$\Phi_{m}(\overset{\to }{r})=\int_{V}\Phi_{x}({\overset{\to }{r}}^{\prime })\eta ({\overset{\to }{r}}^{\prime })G_{m}(\overset{\to }{r},{\overset{\to }{r}}^{\prime })d{\overset{\to }{r}}^{\prime },$$ (5)

where $G_{m}(\vec{r},{\vec{r}}_{s})$ is Green’s function—solution of Eq. (4) when a source term is replaced by a unitary source $\delta (\vec{r}-{\vec{r}}_{s})$. In a special case of an infinite, homogeneous medium, the analytical expression for Green’s function is^27^

$$G(\overset{\to }{r},{\overset{\to }{r}}_{s})=\frac{\exp(-\mu_{\mathrm{eff}}|\overset{\to }{r}-{\overset{\to }{r}}_{s}|)}{4\pi D|\overset{\to }{r}-{\overset{\to }{r}}_{s}|}.$$ (6)

One possible way of modeling the spatial dependence of the optical properties is the implementation of the finite element mesh, where each element is a region of small volume and its specific optical properties. This method is known as the finite element method (FEM), see Sec. 2.2.

### 2.2 Calculation of Fluence by Finite Element Method

The whole space (V) considered is divided into a finite number ($N_{e}$) of elements of finite volume. Every element is defined by its vertices—nodes of the finite element mesh. The elements are usually tetrahedrons or cubes, in this work, we will use tetrahedrons; therefore, every element will have four nodes. Adjacent elements share one triangular face with three common nodes. However, it is also possible that two elements have one shared segment (two common nodes) or even only one shared vertex (one common node). Elements can not have other intersections, apart from a trivial empty set. The fluence Φ in any point ($\vec{r}$) of the medium is approximated by its finite element representation $\Phi^{h}(\vec{r})$, as a weighted sum of fluences in mesh nodes $\Phi_{j}$^30^^–^^32^

$$\Phi (\vec{r})\approx \Phi^{h}(\vec{r})=\sum_{j=1}^{N_{n}}u_{j}(\vec{r})\Phi_{j},$$ (7)

where $N_{n}$ is the number of nodes. The weights $u_{j}(\vec{r})$ depend on the geometrical relations between $\vec{r}$ and node coordinates ${\vec{r}}_{j}$, and they are basis functions which span the whole space V. A function $u_{j}(\vec{r})$ corresponding to node j should be chosen in such a way that it is equal to one in that node, i.e., $u_{j}({\vec{r}}_{j})=1$, and equal to zero in all other mesh nodes, $u_{j}({\vec{r}}_{i})=0$ for i≠j. A set of coordinates $\vec{r}$ where a basis function $u_{j}(\vec{r})$ is different from zero is limited (it is said that these basis functions have limited support).

To solve for the finite element representation of the fluence $\Phi^{h}$, we start from the diffusion equation [Eq. (2)], omitting the subscripts for the wavelength

$$(-\nabla D(\vec{r})\nabla +\mu_{a}(\vec{r}))\Phi^{h}(\vec{r})=q_{0}(\vec{r}).$$ (8)

Multiplying both sides by some function $v(\vec{r})$ (Galerkin method^33^) and integrating over the whole space volume V gives

$$\int_{V}v(\vec{r})[-\nabla D(\vec{r})\nabla +\mu_{a}(\vec{r})]\Phi^{h}(\vec{r})dV=\int_{V}v(\vec{r})q_{0}(\vec{r})dV.$$ (9)

After exploiting mathematical identities and theorems to transform the derivative of the product, volume integral into a surface integral and applying the Robin boundary condition,^27^^,^^34^ Eq. (9) (when $\Phi^{h}(\vec{r})$ is expanded), for specific $v(\vec{r})=u_{i}(\vec{r})$ becomes

$$\overset{N}{\underset{j=1}{\sum }}\{\int_{V}D(\overset{\to }{r})\nabla u_{i}(\overset{\to }{r})\nabla u_{j}(\overset{\to }{r})dV+\int_{V}\mu_{a}(\overset{\to }{r})u_{i}(\overset{\to }{r})u_{j}(\overset{\to }{r})dV+\frac{1}{2A}\oint_{S}u_{i}(\overset{\to }{r})u_{j}(\overset{\to }{r})dS\}\Phi_{j}=\;=\int_{V}u_{i}(\overset{\to }{r})q_{0}(\overset{\to }{r})dV,$$ (10)

where the closed surface S is defined by the boundaries of volume V and A is the effective Fresnel coefficient.^27^^,^^34^ Writing analogous equations for all basis functions $u_{i}(\vec{r})$, $i=1,\ldots ,N_{n}$, the system of equations is obtained^32^

$$(K+C+\frac{1}{2A}B)\cdot \Phi =A\cdot \Phi =Q,$$ (11)

where $K=[K_{ij}]$, $C=[C_{ij}]$, $B=[B_{ij}]$, and $A=[A_{ij}]$ are $N_{n}\times N_{n}$ matrices with entries defined by

$$K_{ij}=\int_{V}D(\overset{\to }{r})\nabla u_{i}(\overset{\to }{r})\nabla u_{j}(\overset{\to }{r})dV;\;C_{ij}=\int_{V}\mu_{a}(\overset{\to }{r})u_{i}(\overset{\to }{r})u_{j}(\overset{\to }{r})dV;\;B_{ij}=\oint_{S}u_{i}(\overset{\to }{r})u_{j}(\overset{\to }{r})dS;\;A_{ij}=K_{ij}+C_{ij}+\frac{1}{2A}B_{ij};$$ (12)

while

$$\Phi =[\begin{array}{cccc} \Phi_{1} & \Phi_{2} & \ldots & \Phi_{N_{n}} \end{array}{]}^{T},$$ (13)

and

$$Q={\left[\begin{array}{cccc} \int_{V}u_{1}(\overset{\to }{r})q_{0}(\overset{\to }{r})dV & \int_{V}u_{2}(\overset{\to }{r})q_{0}(\overset{\to }{r})dV & \ldots & \int_{V}u_{N_{n}}(\overset{\to }{r})q_{0}(\overset{\to }{r})dV \end{array}\right]}^{T}$$ (14)

are $N_{n}\times 1$ column vectors.

Numerical computation of the integrals in Eqs. (12) and (14) is performed within element volumes, exploiting the limited support of basis functions, which results in non-zero integrands only within certain elements. Therefore, all matrices K, C, B and the resulting system matrix A are sparse. For calculating the source vector, depending on the coordinate of the point source ${\vec{r}}_{s}$, the element which contains that source is found (here we consider only point sources; in FEM in general, sources can be distributed as well). The barycentric coordinates of ${\vec{r}}_{s}$ are found with respect to the tetrahedron nodes, and finally, the corresponding integrals are calculated. The fluence vector Φ is computed using fast numerical methods for the inversion of sparse matrices.^7^

To calculate the fluence of the fluorescent light, Eq. (5) is used, where first the fluence $\Phi_{x}$ is computed. The Green’s function $G_{m}(\vec{r},{\vec{r}}^{\prime })$ is computed similarly, just finding $G_{m}({\vec{r}}^{\prime },\vec{r})$ instead, which is computationally faster, because the source is fixed at $\vec{r}$, and the result is the same because of the reciprocity theorem.^35^^–^^37^

Now, we have the methods to solve the forward problem—calculating light fluence at excitation and fluorescent wavelengths at arbitrary positions in the medium, knowing the optical properties.

### 2.3 Inverse Model

We assume to know the optical properties of the background medium—absorption coefficients $\mu_{a}(\vec{r})$ and reduced scattering coefficients $\mu_{s}^{\prime }(\overset{\to }{r})$. Our goal is to reconstruct the fluorescent yield $\eta (\vec{r})$ everywhere in the medium, starting from the fluorescent signal measurements. The measurement data is collected by n interstitially placed optical fibers, and the light is delivered through the same fibers. When light is emitted from one fiber to the medium, the other n−1 are collecting, and that is repeated for every fiber delivering light. In this way, all n(n−1) source-detector pairs are covered, and the light is detected at both—the excitation and the fluorescent wavelengths. To cancel out the experimental uncertainties, such as the source power, or collection efficiency of a fiber, we express the measurements in the form of the normalized Born ratio^38^—the ratio between the signals detected at the fluorescent and the excitation wavelengths, respectively. Therefore, we have $N_{m}=n(n-1)$ measurement points, defined as Born ratios, every measurement point corresponding to one source-detector pair. The forward model, expressed in terms of a vector of finite element mesh nodal values of fluorescent yields, $\eta =(\eta_{1},\eta_{2},\ldots ,\eta_{N_{n}})$, gives the expression for the measurements from the source with index s and the position ${\vec{r}}_{s}$, and the detector with index d, at the position ${\vec{r}}_{d}$^22^^,^^26^

$$F_{s,d}(\eta )=\frac{1}{G_{x}({\overset{\to }{r}}_{d},{\overset{\to }{r}}_{s})}\overset{N_{n}}{\underset{i=1}{\sum }}G_{x}({\overset{\to }{r}}_{i},{\overset{\to }{r}}_{s})G_{m}({\overset{\to }{r}}_{i},{\overset{\to }{r}}_{d})\eta_{i}\Delta V,$$ (15)

where $\eta_{i}$ is the fluorescent yield in node i, with a spatial coordinate ${\vec{r}}_{i}$, ΔV is the element volume, and $G_{x}$ and $G_{m}$ are Green’s functions solutions at the excitation and fluorescent emission wavelengths, respectively, as in Sec. 2.1. These solutions $G_{x,m}({\vec{\rho }}_{d},{\vec{\rho }}_{s})$ are obtained by computing the corresponding fluence at ${\vec{\rho }}_{d}$ when the unitary source is placed at ${\vec{\rho }}_{s}$. Note that this Eq. (15) follows from Eq. (5) when the normalization to the excitation signal is done and after numerical implementation of the integration over all the elements of the finite element mesh.

The arranged pairs (s,d) of source and detector indices are mapped to a set of integer numbers from 1 to $N_{m}$. The mapping function is the following: f(s,d)=d−1+(n−1)(s−1)+p(s,d), where p(s,d)=0 if s<d and p(s,d)=1 if s>d. The forward model vector of the estimated Born ratios is then obtained

$$F(\eta )=(F_{1}(\eta ),\ldots ,F_{i}(\eta ),\ldots ,F_{N_{m}}(\eta )),\;\mathrm{where}\;i=f(s,d).$$ (16)

The same mapping is done on measurement points, creating the measurement vector M, which contains all $N_{m}$ Born ratios of the detected fluorescent and excitation light signals. The discrepancy between the measurements and the forward model is the error vector δ(η)=M−F(η).

The inverse problem can be formulated as estimating the vector of fluorescent yields η such that the error vector δ(η) is minimal in some metrics. A standard metrics is the Euclidean or 2-norm, which we denote as ‖δ‖. The number of unknowns $N_{n}$ (components of vector η) is usually much greater than the number of measurements $N_{m}$ ($N_{n}∼10^{4}\gg N_{m}∼10^{2}$), so the problem is ill-posed, and a regularization should be added to its formulation. A common approach is Tikhonov regularization,^32^^,^^39^ where a quadratic term with the 2-norm of the vector of unknowns is included in the objective function. The goal is to minimize the following^22^^,^^26^

$$\Omega (\eta )=\|M-F(\eta ){\|}^{2}+\lambda {\left\|L(\eta -\eta_{0})\right\|}^{2},$$ (17)

where λ is the regularization parameter, L is the regularization matrix (dimensions $N_{n}\times N_{n}$), and $\eta_{0}$ is the initial estimate (guess) for the vector of unknowns η.

If η is the parameter that minimizes the cost function Ω(η), then the first derivative $\frac{\partial \Omega (\eta )}{\partial \eta }$ is equal to zero, which implies

$$-2{\frac{\partial F(\eta )}{\partial \eta }}^{T}(M-F(\eta ))+2\lambda L^{T}L(\eta -\eta_{0})=0.$$ (18)

Defining the Jacobian as $N_{m}\times N_{n}$ matrix $J=\frac{\partial F(\eta )}{\partial \eta }$, we can write

$$J^{T}(M-F(\eta ))=\lambda L^{T}L(\eta -\eta_{0}).$$ (19)

If $\eta_{i}$ and $J_{i}$ are the estimates vector and the Jacobian of iteration i, then, the forward model calculated for the iteration i+1 can be, using the Taylor expansion, approximated as

$$F(\eta_{i+1})\approx F(\eta_{i})+J_{i}(\eta_{i+1}-\eta_{i}),$$ (20)

where it is assumed that the estimates vectors of successive iterations $\eta_{i+1}$ and $\eta_{i}$ are close enough to have a good approximation by keeping just the first order term. Rewriting Eq. (19) for the next iteration $\eta_{i+1}$ while exploiting the forward model linearization around $\eta_{i}$ in Eq. (20) gives

$$J_{i}^{T}(M-F(\eta_{i})-J_{i}(\eta_{i+1}-\eta_{i}))=\lambda L^{T}L(\eta_{i+1}-\eta_{0}),$$ (21)

which can be rewritten as

$$J_{i}^{T}\delta (\eta_{i})-J_{i}^{T}J_{i}(\eta_{i+1}-\eta_{i})=\lambda L^{T}L(\eta_{i+1}-\eta_{i})+\lambda L^{T}L(\eta_{i}-\eta_{0}).$$ (22)

Finally, the update equation for the fluorescent yield estimates vector is^22^

$$\eta_{i+1}=\eta_{i}+(J_{i}^{T}J_{i}+\lambda L^{T}L{)}^{-1}(J_{i}^{T}\delta (\eta_{i})-\lambda L^{T}L(\eta_{i}-\eta_{0})).$$ (23)

Equation (23) is general, and in this work, we apply a modified Levenberg-Marquardt algorithm (similar to Axelsson et al.^22^ and Dehghani et al.^32^) where the update equation is

$$\eta_{i+1}=\eta_{i}+(J_{i}^{T}J_{i}+\lambda_{i}L^{T}L{)}^{-1}J_{i}^{T}\delta (\eta_{i}),$$ (24)

with λ being initialized to the maximum of the diagonal of the Hessian matrix: $\lambda_{0}=\max\{\mathrm{diag}(J_{0}^{T}J_{0})\}$ and updated in every iteration to $\lambda_{i}=\max\{\mathrm{diag}(J_{i}^{T}J_{i})\}\cdot 10^{-i/4}$. A more detailed discussion about the regularization follows in Sec. 2.4.

The iterative procedure of updating η stops when the relative decrease of the norm of the error vector ‖δ‖ is <2% or the predefined maximal number of iterations (around 20) is reached.^32^

### 2.4 Regularizations

The solution obtained from the inverse model is regularized and does not have the minimum norm of the error vector. However, it has some properties that are imposed by the choice of the regularization matrix—which can make it “smoother” (less abrupt spatial variations of the reconstructed fluorescent yield) or have similar (very close) values in predefined regions or even very sharp transitions between the regions.

The default regularization applied in this paper is without prior information about the geometry, setting the regularization matrix of the Tikhonov regularization to an identity matrix L=I. This adds some “inertia” on the main diagonal of the Hessian matrix $J^{T}J$, which provides numerical stability when computing inverses of large-dimensional matrices. As a consequence, the solution appears “smooth” in space.

It is possible to include a priori knowledge about the geometry of the problem, by assuming there are regions in space (for example different tissue types) that should have similar levels of PS drug accumulation—for example, inside the tumor the highest, around the tumor lower, background the lowest. If we are not reducing the number of unknowns of the original inverse problem, thus permitting different values of reconstructed fluorophore absorption coefficients within the regions, the geometrical biasing of the solution is called soft priority, and the entries of the regularization matrix $L=[L_{ij}]$ are defined as^22^^,^^40^

$$L_{ij}=\{\begin{array}{ll} 1, & \text{if }i=j;\\ \frac{-1}{N_{R(i)}}, & \text{else if nodes }i\text{ and }j\text{ belong to the same region }R(i)\equiv R(j)\text{ with }N_{R(i)}\text{ nodes};\\ 0, & \text{otherwise}. \end{array}$$ (25)

Suppose we restrict to only a few possible values for the reconstructed fluorophore absorption coefficients, i.e., divide the whole geometry of the medium into $N_{r}$ non-overlapping regions, where all nodes within a particular region have the same reconstructed value. In that case, the number of unknowns is reduced to $N_{r}$, and the geometrical biasing of the solution is called hard prioring. When it is reasonable to assume homogeneous properties within the regions of the tissue, this regularization results in quantitatively more accurate reconstruction, as reported by Dehghani et al.^41^ and Srinivasan et al.^42^ The main idea underlying this method will be applied in S2, where the results obtained without any geometrical biasing in S1 will be used to define the regions in S2 (see Sec. 2.5).

It is, of course, possible to apply any regularization different from Tikhonov, and some examples are given in the work by Jagannath and Yalavarthy^43^ and in the review by Okawa and Hoshi.^44^

### 2.5 Second Stage of the Reconstruction (S2)

The inverse model provides the solution for the fluorescent yields in all nodes, using regularization and the Levenberg-Marquardt iterative algorithm, and we refer to this method as S1. The solution obtained at this stage is usually smoother than it should be (obtained fluorescent yields do not show abrupt spatial variations, because of the regularization) and therefore has a quantitatively larger error. This is more evident in homogeneous regions, where almost the same fluorophore absorption coefficient is expected over the whole volume of the region. This is the key assumption for S2. Assuming the entire medium can be divided into $N_{r}$ non-overlapping regions (sets of finite element mesh nodes) of homogeneous fluorophore absorption coefficients, the inverse problem to solve becomes more straightforward—the number of unknowns is reduced to $N_{r}$, while still having $N_{m}$ measurement points. Usually, $N_{r}\ll N_{m}$.

**Fig. 1:**
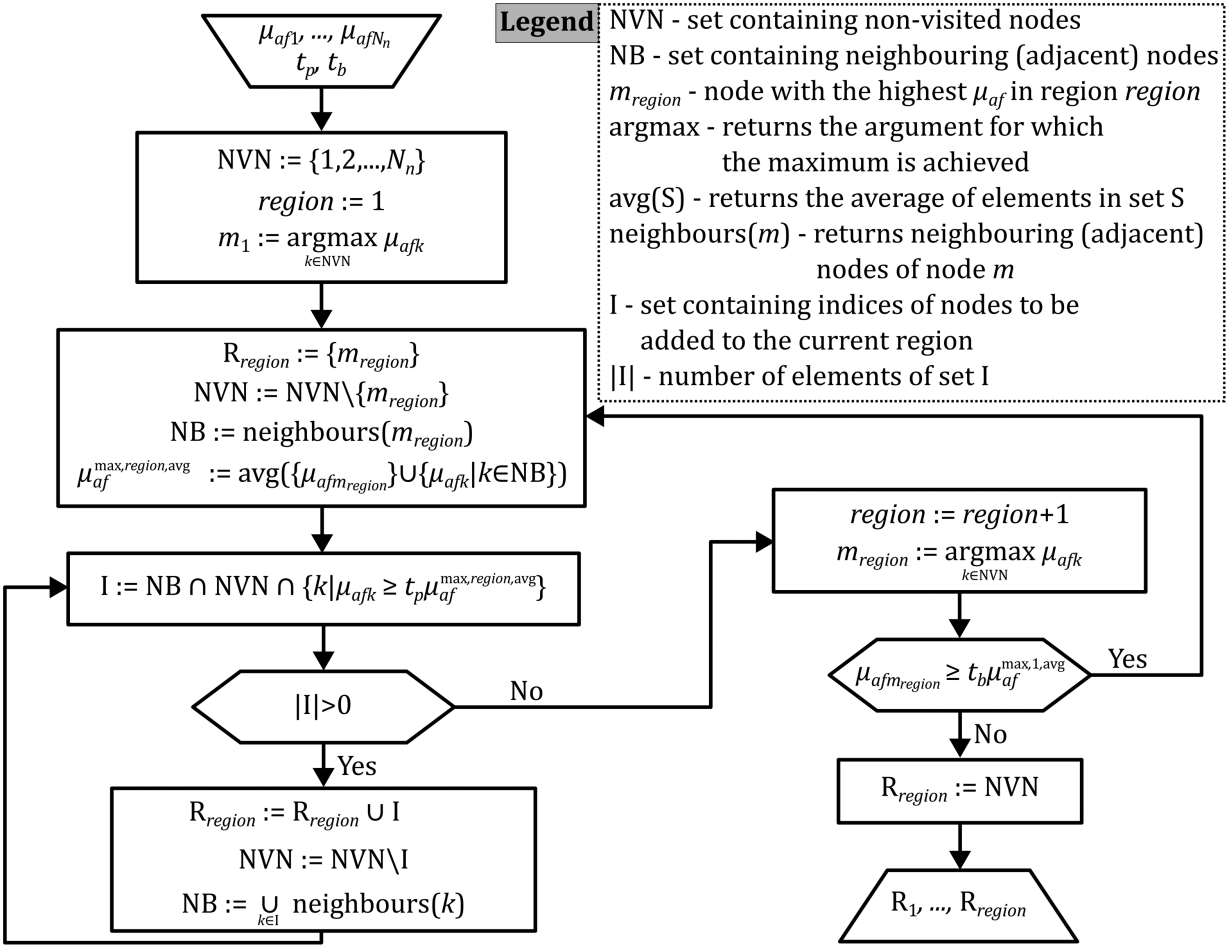
Algorithm for defining the regions of homogeneous fluorophore absorption $R_{1},\ldots ,R_{N_{r}}$ based on inputs from S1—reconstructed fluorophore absorptions in all nodes $\mu_{af1},\ldots \mu_{afN_{n}}$, for chosen thresholds $t_{p}$, $t_{b}$.

The regions are defined after S1, by looking at the obtained spatial distribution of fluorescent yields, i.e., fluorophore absorption coefficients. The values of fluorophore absorptions in the mesh nodes ($\mu_{af1},\mu_{af2},\ldots ,\mu_{afN_{n}})$ are compared with thresholds. For the two finite element mesh nodes belonging to the same element (tetrahedron), we say they are adjacent or neighboring. Starting from the mesh node $m_{1}$ with the highest fluorophore absorption coefficient in the medium, the first region is initialized as the set containing only that node. The average fluorophore absorption coefficient of this node and all its neighboring nodes is $\mu_{af}^{\max,1,\mathrm{avg}}$. In the next iteration, this region can be expanded by including all the neighboring nodes of the node $m_{1}$, which have fluorophore absorption greater than or equal to the threshold $t_{p}\mu_{af}^{\max,1,\mathrm{avg}}$, where $0<t_{p}<1$ is the given fraction that determines the peak threshold. This procedure is repeated as long as there are neighboring nodes (k) of any of the nodes from the first region, not yet included in the first region, which satisfy the condition $\mu_{afk}\geq t_{p}\mu_{af}^{\max,1,\mathrm{avg}}$. After the iteration in which all the adjacent nodes, not yet included in the first region, had fluorophore absorption below the threshold $t_{p}\mu_{af}^{\max,1,\mathrm{avg}}$, the first region is concluded. Then, in case there is a node $m_{2}$ with the highest fluorophore absorption coefficient among all nodes outside of the first region, satisfying the condition $\mu_{{\mathrm{afm}}_{2}}\geq t_{b}\mu_{af}^{\max,1,\mathrm{avg}}$, for the given background threshold fraction $0<t_{b}<1$, the second region is initialized as the set containing only that node ($m_{2}$). The average fluorophore absorption coefficient of this node and all its neighboring nodes is $\mu_{af}^{\max,2,\mathrm{avg}}$. The iterative procedure for including neighboring nodes continues, now with the threshold $t_{p}\mu_{af}^{\max,2,\mathrm{avg}}$, defining the second region. In case there is a node ($m_{3}$) in the finite element mesh which is not included in the first two regions, and still satisfies the condition $\mu_{{\mathrm{afm}}_{3}}\geq t_{b}\mu_{af}^{\max,1,\mathrm{avg}}$, the next region is defined, and so on. The first time the highest value of $\mu_{af}$ outside of the regions defined so far is below the threshold $t_{b}\mu_{af}^{\max,1,\mathrm{avg}}$, the number of regions is concluded by ascribing all the remaining nodes to the background region. The flowchart diagram of this algorithm is shown in Fig. 1.

The transition matrix T (also called a priori matrix^41^), with dimensions $N_{n}\times N_{r}$, provides the mapping between the original vector $\eta^{\left(1\right)}$ of $N_{n}$ fluorescent yields, as a result of S1, and the vector of unknowns $\eta^{\left(2\right)}$ in S2, which has only $N_{r}$ components. A matrix entry $T_{ij}$ is equal to 1 if the node i belongs to region j; otherwise, it is equal to 0. The relations between $\eta^{\left(1\right)}$ and $\eta^{\left(2\right)}$, as well as between the reduced (S2) Jacobian $J^{\left(2\right)}$ and the full (S1) Jacobian $J^{\left(1\right)}$ are

$$\eta^{\left(1\right)}=T\eta^{\left(2\right)},\;J^{\left(2\right)}=J^{\left(1\right)}T.$$ (26)

The inverse problem is now solved without regularization, by applying the Gauss-Newton^45^^,^^46^ iterative method with the updated equation for $\eta_{i}^{\left(2\right)}$

$$\eta_{i+1}^{\left(2\right)}-\eta_{i}^{\left(2\right)}=((J^{\left(2\right)}{)}_{i}^{T}J_{i}^{\left(2\right)}{)}^{-1}(J^{\left(2\right)}{)}_{i}^{T}\delta (\eta_{i}^{\left(1\right)}).$$ (27)

The initial guess for the vector $\eta_{0}^{\left(2\right)}$ is the vector of averages of the fluorescent yields in regions, obtained from S1, thus not expecting to be too far from the “solution” (value of η for which ‖δ(η)‖ is minimal). Because the system of equations is now overdetermined, the forward model in this stage cannot match all the measurement points but provides a smaller error ‖δ‖ than in S1. This error is evaluated for different divisions of the medium into regions, by changing the threshold fractions $t_{p}$ and $t_{b}$. A pair of threshold fractions $\left(t_{p},t_{b}\right)$ is given as input to the regionalization algorithm defined in Fig. 1. The output of the regionalization algorithm is the array of regions $R_{1},R_{2},\ldots ,R_{N_{r}}$. The regionalization algorithm is executed for different threshold pairs $\left(t_{p},t_{b}\right)$, resulting in different divisions of the medium into regions, i.e., different arrays $R_{1},R_{2},\ldots ,R_{N_{r}}$. The regionalization (division) for which the error $\left\|\delta (\eta^{\left(2\right)})\right\|$ is the smallest, and the corresponding vector $\eta^{\left(2\right)}$, are the final results of S2.

## 3 Materials and Methods

### 3.1 Computer Implementation

A finite element mesh is defined over the considered inhomogeneous medium, and the FEM is used to compute the forward model fluences and normalized Born ratios in Eq. (15). The mesh elements are tetrahedrons, and the mesh creation and all FEM computations are done in MATLAB (The MathWorks Inc., 2022) using the open-source NIRFAST package.^32^^,^^47^ Mesh nodes are placed more densely around the fiber tip positions to capture more dynamic spatial variations of fluence near the light sources and detectors. The resulting meshes had from 20,000 to 70,000 nodes, and between 90,000 and 320,000 elements, with an average size of around $(5\times 5\times 5)\;{\mathrm{cm}}^{3}$. The mesh, used to calculate the forward model, is called the forward mesh. Details about the forward meshes used in this work are given in Table 1. The reconstruction mesh, used to solve the inverse problem, has a lower resolution (for example 6480 nodes) to reduce the computational cost and improve the numerical stability of the Hessian matrix inversion. The interpolation from the forward mesh onto the reconstruction mesh and vice versa is done in every iteration of the reconstruction procedure: the forward model and the corresponding Jacobian are first computed on the forward mesh, then the dimension of the problem is reduced by interpolating nodal values from higher resolution forward to lower resolution reconstruction mesh, on which the update equation [Eq. (24)] is solved, and the obtained nodal values are extrapolated back from the reconstruction to the forward mesh. Mesh resolution will affect the quality of the reconstruction and the time needed for the convergence. One should find a balance between the number of measurement points, the number of unknowns (size of the forward mesh), the computational power, the regularization, the accuracy of the forward model, and finally, the solution.

**Table 1:** Finite element meshes used in this work, with the details about the size, numbers of nodes, and elements.

Mesh	Size (mm × mm × mm)	Number of nodes	Number of elements
1	52 × 57 × 40	28,296	130,124
2	52 × 57 × 40	64,325	312,676
3	40 × 50 × 34	49,921	237,041
4	40 × 50 × 34	20,745	92,705
5	50 × 50 × 40	49,900	240,333
6	50 × 50 × 40	22,972	104,406

For S2, adaptive thresholds were implemented—the threshold fractions $t_{p}$ and $t_{b}$ from Sec. 2.5 are varied, resulting in the one providing the lowest final ∥δ∥. This takes more computational time but is more robust than the approach with fixed thresholds. In the current implementation, it is assumed $t_{p}=t_{b}$, and nine equidistant values for the threshold fractions are taken from the interval (0.33, 0.87).

**Fig. 2:**
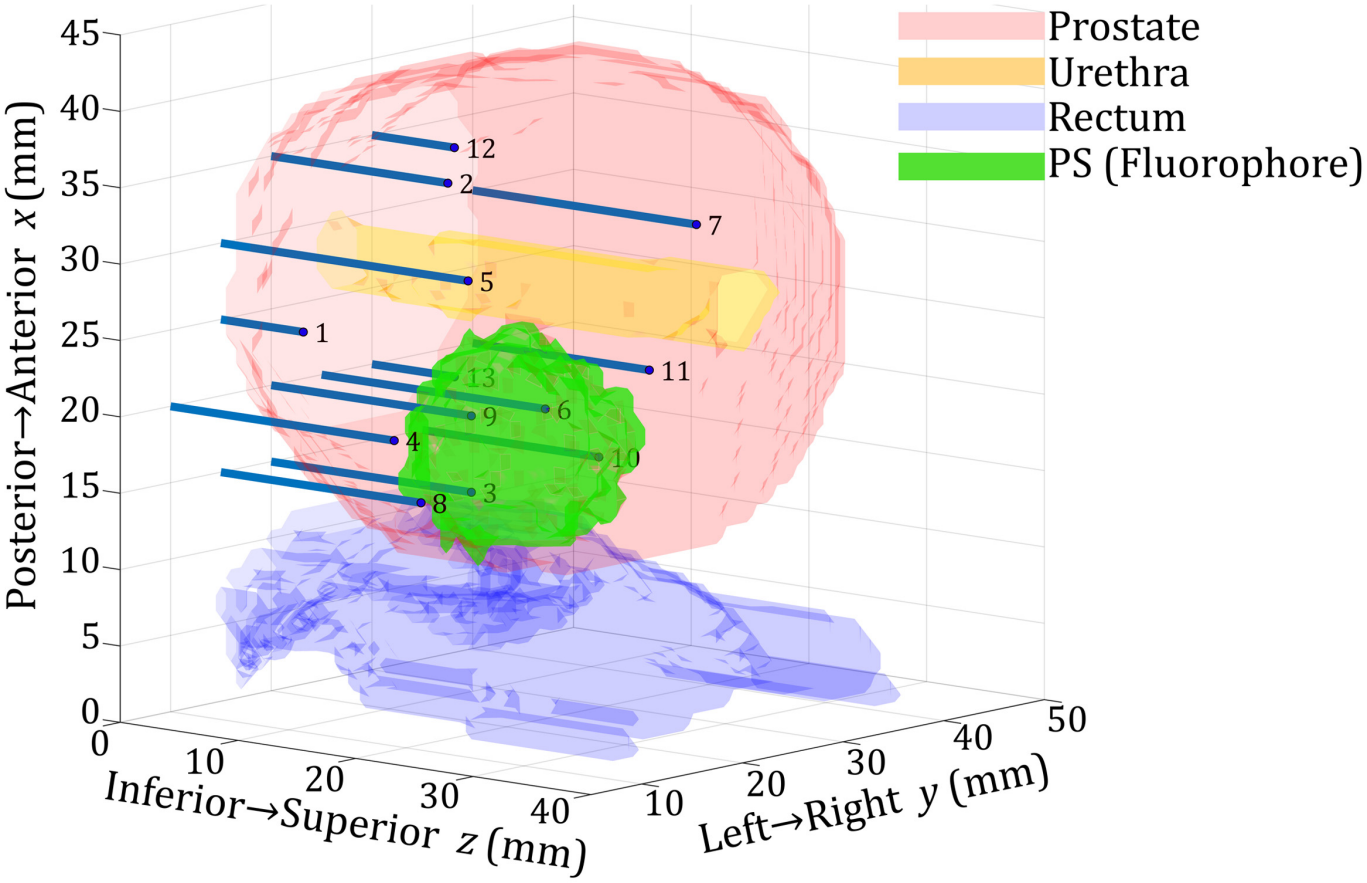
3D model of prostate tissue (semi-transparent red) with the urethra (semi-transparent yellow), rectum (semi-transparent blue), and fluorescent PS (green). Blue lines and blue circles represent fibers and their tips (labeled with numbers), respectively. PS distribution shown is a result of the reconstruction from the simulation with default parameters (see Sec. 3.3) and without noise. The same Mesh 1 (see Table 1) was used for data generation and inverse problem solving. A coarser 6480-node mesh was used for matrix inversion. 3D models of the prostate, urethra, and rectum obtained from clinical ultrasound data. Labels on axes x, y, and z correspond to the sides and directions of the patient’s body.

The graphical representation of the reconstructed fluorophore absorption relies on the mapping from the forward mesh nodal values to the values in arbitrary positions in space, where every point in space is within an element, and its barycentric coordinate within the element determines the contribution of each of the element nodes. The graphical representation is a 2-dimensional (2D) color map of fluorophore absorption coefficients in a single xy-plane (the chosen slice perpendicular to the z-axis, see Figs. 4, 5, 7, 8, 9, and 11). Note that even the homogeneous GT fluorophore distributions will appear in different colors, because of this mapping between the FEM mesh and 2D graphics mesh. Blue circles denote fiber tip projections in xy-plane. After S2, the space is divided into a few regions of homogeneous fluorophore absorption coefficients with sharp borders. It is possible to provide a 3-dimensional (3D) representation of the regions using semi-transparent boundary surfaces [see Figs. 2 and 10(e)]. This way, the estimates of the reconstructed inclusion (region) volumes are also given.

### 3.2 Prostate Anatomy and Interstitial Fiber Placement

In Fig. 2, we give a 3D model of relevant tissues, fiber positions, and the reconstructed fluorophore distribution in space. The reconstruction was performed on simulated data, following the default scenario described in Sec. 3.3, without noise. The same finite element mesh was used for data generation and reconstruction. This graphical representation should help the reader to understand the geometry of the interstitial fiber placement. Fiber coordinates (see Table 2) and 3D models of the prostate, urethra, and rectum are obtained from clinical ultrasound data, as in our previous work.^26^ The fiber configuration in a particular PDT treatment is the result of Cimmino’s optimization method, where the minimum threshold light dose is delivered to the target volume while minimizing the surrounding tissue exposure.^8^^,^^48^

**Table 2:** Fiber coordinates used in simulations.

Fiber number	1	2	3	4	5	6	7	8	9	10	11	12	13
x (mm)	25	35	15	20	30	20	30	15	20	15	20	35	20
y (mm)	15	20	20	10	15	25	40	15	20	35	40	30	30
z (mm)	7	15	17	19	21	19	19	17	17	15	15	7	7

### 3.3 Numerical Simulations

Computer codes for numerical simulations, including data creation, reconstruction, and graphical representation, were implemented in MATLAB. Simulated measurement data were created by applying the forward model FEM, described in Sec. 3.1.

In all simulated scenarios, a single-spherical inclusion was considered. Unless other specified, the following parameters were used as default. GT background fluorophore absorption $\mu_{afbg,gt}=0.01\;{\mathrm{cm}}^{-1}$, GT inclusion fluorophore absorption $\mu_{afi,gt}=0.10\;{\mathrm{cm}}^{-1}$, reduced scattering coefficient $\mu_{s}^{\prime }=8.7\;{\mathrm{cm}}^{-1}$, and absorption coefficient of the background $\mu_{a}=0.50\;{\mathrm{cm}}^{-1}$. The inclusion was centered at $(x_{C},y_{C},z_{C})=(19,25,18)\;\mathrm{mm}$, with radius R=6.7  mm. A total of 13 fibers were placed^26^ at the positions given in Table 2 and shown in Fig. 2.

### 3.4 Evaluation of the Reconstruction

To quantitatively evaluate the performance of the reconstruction, here, we introduce the following relative errors:

• reconstructed volume of the inclusion, relative error $\delta V_{i}$;
• reconstructed fluorophore absorption coefficient of the inclusion, relative error $\delta \mu_{\mathrm{afi}}$;
• reconstructed fluorophore absorption coefficient of the background, relative error $\delta \mu_{\mathrm{afbg}}$;
• average absolute value of the relative error of the reconstructed fluorophore absorption coefficient $\left\langle |\delta \mu_{af}(\overset{\to }{r})|\right\rangle$ in the whole medium (shorter ⟨|e|⟩).

Note that it only makes sense to define relative errors $\delta V_{i}$, $\delta \mu_{\mathrm{afi}}$, and $\delta \mu_{\mathrm{afbg}}$ for the results of the second stage of the reconstruction, because these parameters refer to the regions defined only in that stage. On the other hand, the absolute value of the relative error |e| can be defined for every node, and we will compare the average in the whole medium ⟨|e|⟩ after S1 and after S2.

### 3.5 Phantoms

Tissue-mimicking phantoms were realized to validate the proposed model. A hybrid approach of combining solid and liquid phantoms similar to the work by Ghauri et al.^26^ was followed. The liquid solution used for background optical properties of the tissue phantom is a water solution of Intralipid (200  mg/ml, Fresenius Kabi, Ltd., Germany) and India ink (a - Rotring, Germany; and b - Higgins, 44201 Chartpak Inc., USA). India ink (a) was first diluted in water to 1%, then 4.1 ml of this stock solution was added to a solution containing 35.4 ml of intralipid (20%w/v) and 960 ml of water, mixed with a magnetic stirrer to achieve homogeneous properties: absorption coefficient $\mu_{ab}=0.24\;{\mathrm{cm}}^{-1}$ and the reduced scattering coefficient $\mu_{sb}^{\prime }=8.3\;{\mathrm{cm}}^{-1}$ (both at 690 nm). The optical properties were chosen to mimic those of a human prostate.^49^ The solid inclusions were realized as spheres of radius R=5.5  mm. The material used was water-based gelatin with the addition of proper amounts of Intralipid and India ink (b) to achieve the absorption coefficient $\mu_{a}=0.20\;{\mathrm{cm}}^{-1}$ and $\mu_{s}^{\prime }=15.7\;{\mathrm{cm}}^{-1}$. The same protocol as in our previous work^26^ was applied to make these phantoms. The PS used was verteporfin, an active substance of the drug Visudyne (Cheplapharm Arzneimittel GmbH, Germany). This PS is excited at around 690 nm and re-emits fluorescent light at wavelengths longer than 700 nm. Solid inclusions were realized with different concentrations of verteporfin, ranging from 0.3 to 1.8  mg/kg. Optical characterization of bulk properties of both solid and liquid phantom components was done using a time-domain diffuse optical spectroscopy system.^50^

### 3.6 Experimental Setup

The measurements were performed using SpectraCure’s P18 system.^8^^,^^18^ The system consists of a light delivery unit with 18 photonics modules. Each photonics module (PM) has a laser diode (690 nm), and two detectors—the first detecting excitation light (wavelengths below 700 nm) and the other detecting fluorescence or infrared light (wavelengths above 700 nm). Each PM is SC-connected to a 45-cm-long patch fiber with a core diameter of 400  μm and NA=0.37. Each patch fiber is further SMA-connected to a 200-cm-long silica fiber with the same core diameter and NA=0.22. This fiber, inserted through a brachytherapy needle, has its bare end (tip) free in the tissue phantom (around 2 mm beyond the needle tip) to deliver and collect light, although not simultaneously. PMs are controlled by SpectraCure’s Interactive Dosimetry by Sequential Evaluation (IDOSE) software^8^ for how long the light will be sent or collected. We used n=12 PMs, and therefore, 12 fibers were placed in the tissue phantom, through brachytherapy needles, which were supported at pre-determined positions (holes) in the brachytherapy template (horizontal plane) and specific pre-determined depths (along the vertical axis). The fiber arrangement has been decided based on different simulations of real measurements (see Figs. 8 and 9), such that the chosen configuration, reported in Table 3, results in enough signals above the cut-off threshold (Sec. 4.1), for all tested fluorophore concentrations.

**Table 3:** Another fiber configuration used in simulations and in experiments.

Fiber number	1	2	3	4	5	6	7	8	9	10	11	12
x (mm)	15	20	15	20	25	30	20	25	30	35	30	35
y (mm)	30	35	20	25	30	35	15	20	25	30	15	20
z (mm)	25	15	15	20	20	25	25	15	25	15	15	25

**Fig. 3:**
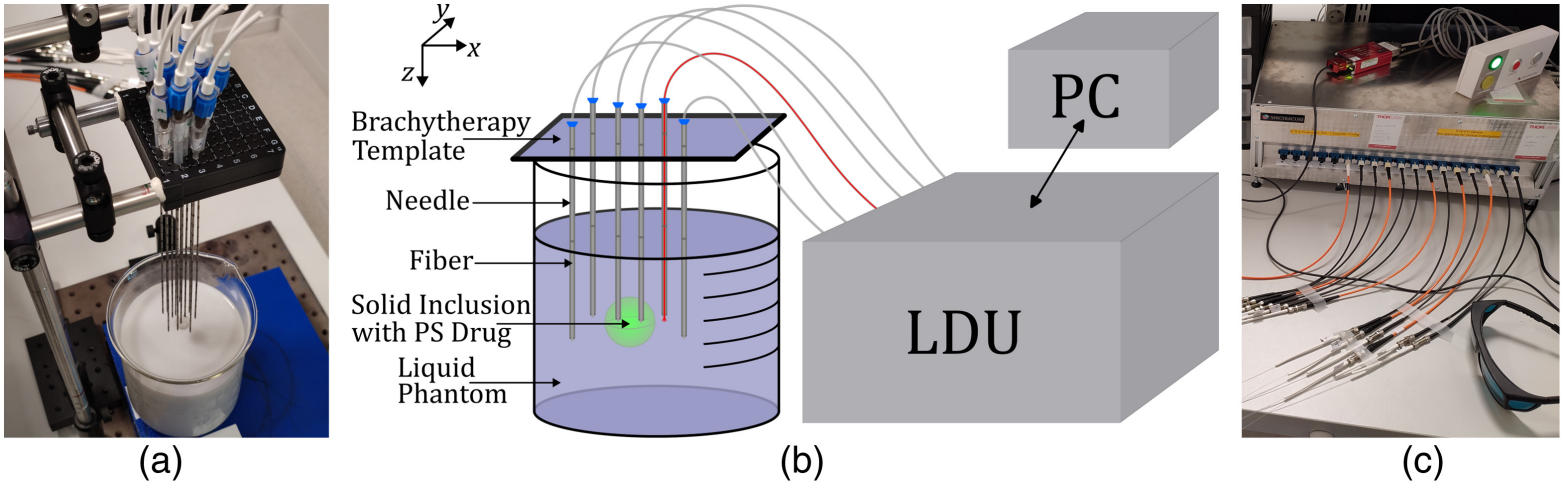
(a) Gelatin sphere attached to two brachytherapy needles (corresponding to fibers 4 and 5) before immersing into a liquid background. (b) Schematics of the laboratory setup: LDU—light delivery unit, and PC—personal computer, with the software sending instructions to and receiving the data from LDU. (c) SpectraCure’s P18 LDU with optical fibers connected to its ports.

By controlling all 12 PMs, tomographic data acquisition is possible: while one fiber is delivering light, the other 11 are collecting—both excitation (690 nm) and infrared fluorescence emission—and this is repeated such that all 12 fibers deliver light, one at a time (monitoring sequence). There are usually several monitoring sequences (3 to 5), during the session, one after another, to follow the PS drug concentration evolution over time (which is of interest in a dynamic environment, such as in vivo during PDT). After each monitoring sequence, a tomographic reconstruction of the fluorophore absorption can be performed. The tissue phantom (Sec. 3.5) is a liquid solution, with the addition of solid fluorophore inclusions suspended by brachytherapy needles. All the fibers and needles are vertical and immersed in the liquid background, only two of the fiber tips are positioned inside the solid fluorophore inclusions. The experimental setup is shown in Fig. 3.

## 4 Results and Discussion

### 4.1 Simulation Study on the Effect of Different Parameters

**Fig. 4:**
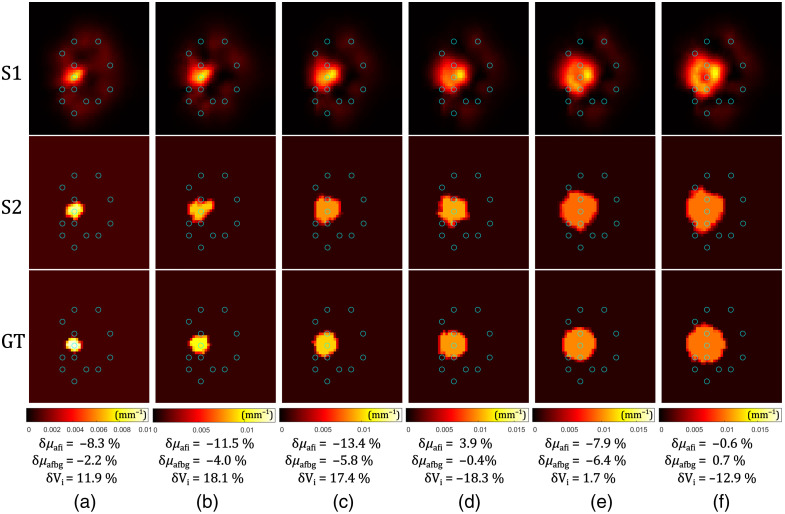
Reconstruction of spherical fluorophore inclusions of different simulated sizes (R): (a) 3 mm, (b) 4 mm, (c) 5 mm, (d) 6 mm, (e) 7 mm, and (f) 8 mm. The results after the S1 reconstruction are in the first row, followed by the S2 reconstruction in the second row, and finally compared with the GT in the third row. The plane cut is perpendicular to the z-axis, through the center of the inclusion sphere. Fluorophore absorption coefficients are shown with different colors (color bars below the GT images). Blue circles represent fiber positions. Relative errors for the reconstructed fluorophore absorption coefficients of the inclusions $\delta \mu_{afi}$ and the background $\delta \mu_{afbg}$, and the volumes of the inclusions $\delta V_{i}$, are given below the images. Data were generated using the denser Mesh 2, whereas the inverse problem was solved using Mesh 1 (see Table 1), with a coarse 6480-node mesh used for matrix inversion.

In simulations, we first examine how the size of the inclusion affects the reconstruction of relative errors. The parameter varied here is the radius of the spherical inclusion (R). The reconstructions after S1 and after S2, are compared with the GT fluorophore absorption distribution, as shown in Fig. 4. Data were generated on the mesh different from the one used for the reconstruction (see the caption of Fig. 4).

For all the considered sphere sizes, relative errors of the reconstructed fluorophore absorption coefficients of the inclusion and the background are always at similar levels (of the order of (1 to 10)%). The absolute value of the relative error of the reconstructed volume is always <20%, which corresponds to about 6% error in linear dimensions (radius or diameter). This can be ascribed to the finite element mesh limitations and differences between the mesh used for data creation and the mesh used for the reconstructions (see the caption of Fig. 4). Considering the colors in Fig. 4, it is possible to observe that the spatial sensitivity is dependent on the fiber positions—S1 reconstruction resulted in some detectable fluorophore absorption (dark red blurs) around the fibers, whereas the rest of the background resulted in values close to 0 (black). S2 of the reconstruction compensates for these artefacts taking advantage of the homogeneous regions assumption. The reconstructed background has a maximum error of <10%. A clear evidence of the benefits of the two-stage reconstruction approach is the following: an average absolute value of the relative error of the reconstruction ⟨|e|⟩ after S1 was always around 90%, whereas this average is drastically reduced after adding S2, resulting in ⟨|e|⟩ in range (1 to 8)%.

It is interesting to note that the introduction of S2 in the reconstruction algorithm has a similar effect on the background artifact removal and the spatial confinement of the reconstructed inclusion as the quadratic source term in the diffusion equation for fluorescence in the case of upconverting nanoparticles.^51^

**Fig. 5:**
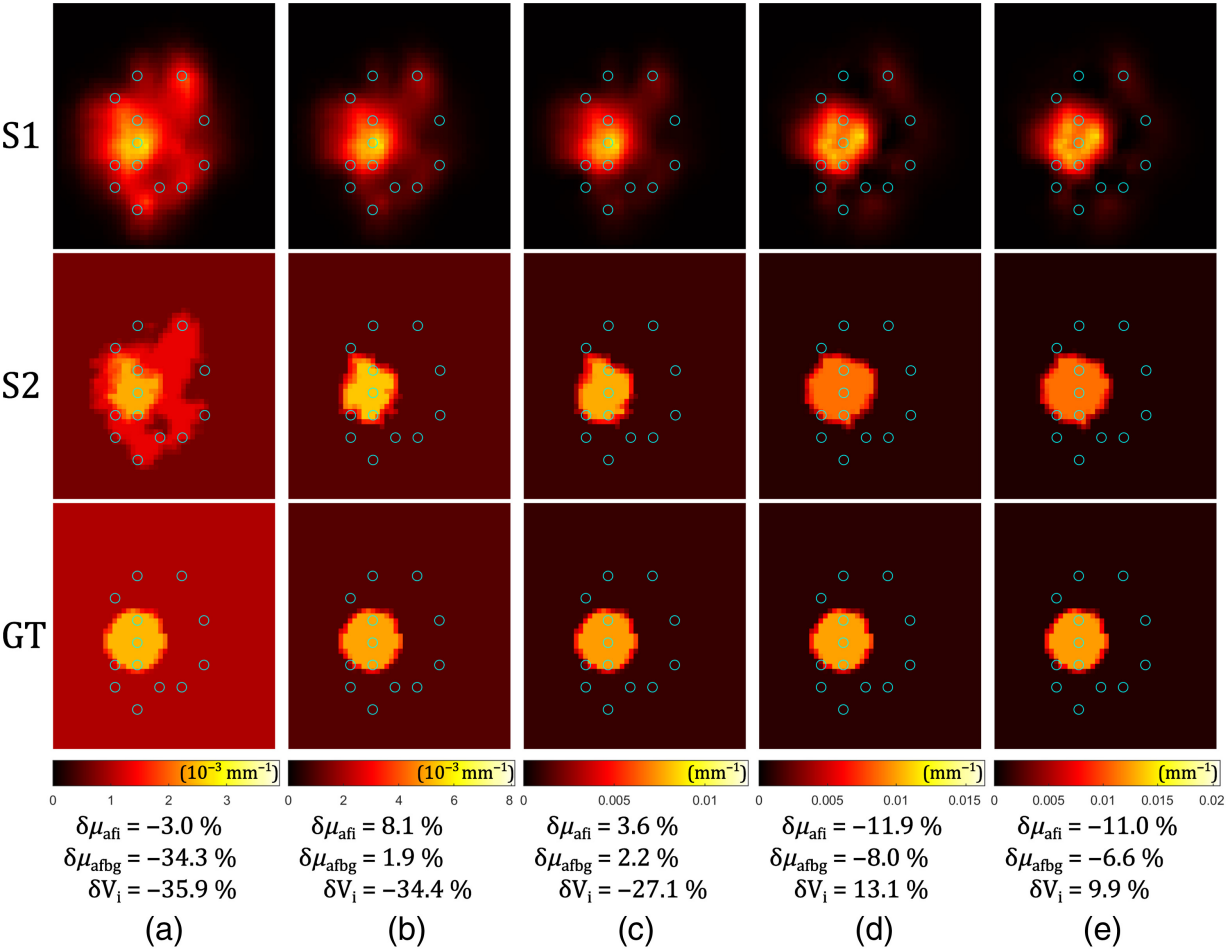
Reconstruction of spherical (R=6.7  mm) inclusions of different simulated fluorophore absorption coefficients ($\mu_{afi}$): (a) $0.025\;{\mathrm{cm}}^{-1}$, (b) $0.050\;{\mathrm{cm}}^{-1}$, (c) $0.075\;{\mathrm{cm}}^{-1}$, (d) $0.100\;{\mathrm{cm}}^{-1}$, and (e) $0.125\;{\mathrm{cm}}^{-1}$. The results after the S1 reconstruction are in the first row, followed by the S2 reconstruction in the second row, and finally compared with the GT in the third row. The plane cut is perpendicular to the z-axis, through the center of the inclusion sphere. Fluorophore absorption coefficients are shown with different colors (color bars below the GT images). Blue circles represent fiber positions. Relative errors for the reconstructed fluorophore absorption coefficients of the inclusions $\delta \mu_{afi}$ and the background $\delta \mu_{afbg}$, and the volumes of the inclusions $\delta V_{i}$, are given below the images. Data was generated using the denser Mesh 2, whereas the inverse problem was solved using Mesh 1 (see Table 1), with a coarse 4590-node mesh used for matrix inversion.

The second varied parameter is the fluorophore absorption coefficient of the inclusion ($\mu_{\mathrm{afi}}$), which is directly proportional to the concentration of the fluorophore. Figure 5 shows the reconstruction results for different ratios of $\mu_{\mathrm{afi}}/\mu_{\mathrm{afbg}}$, where the background fluorophore absorption was kept constant, $\mu_{\mathrm{afbg}}=0.010\;{\mathrm{cm}}^{-1}$. Data were generated on the mesh different from the one used for the reconstruction (see the caption of Fig. 5).

We see that as the inclusion’s fluorophore absorption coefficient increases, the reconstructed volume of the inclusion is more accurate. When the volume is underestimated, the absorption coefficient is overestimated and vice versa. Relative errors of the reconstructed fluorophore absorption coefficients of the inclusion and the background are always at similar levels (of the order of (1 to 10)%). When the fluorophore absorption of the inclusion is similar to the background [Fig. 5(a), $\mu_{\mathrm{afi}}/\mu_{\mathrm{afbg}}=2.5$], the reconstruction S2 resulted in three regions, and therefore, a larger error for the reconstructed background, as the region between the background and the inclusion was a “transitional” region with the reconstructed fluorophore absorption coefficient 32% higher than the GT background. In this particular case, the average absolute value of the relative error ⟨|e|⟩ after S2 was 34%, whereas after S1, it was 89%. In all other cases, the initial ⟨|e|⟩ of around 90% after S1 was reduced to a value in the interval (2 to 10)% by adding S2.

**Fig. 6:**
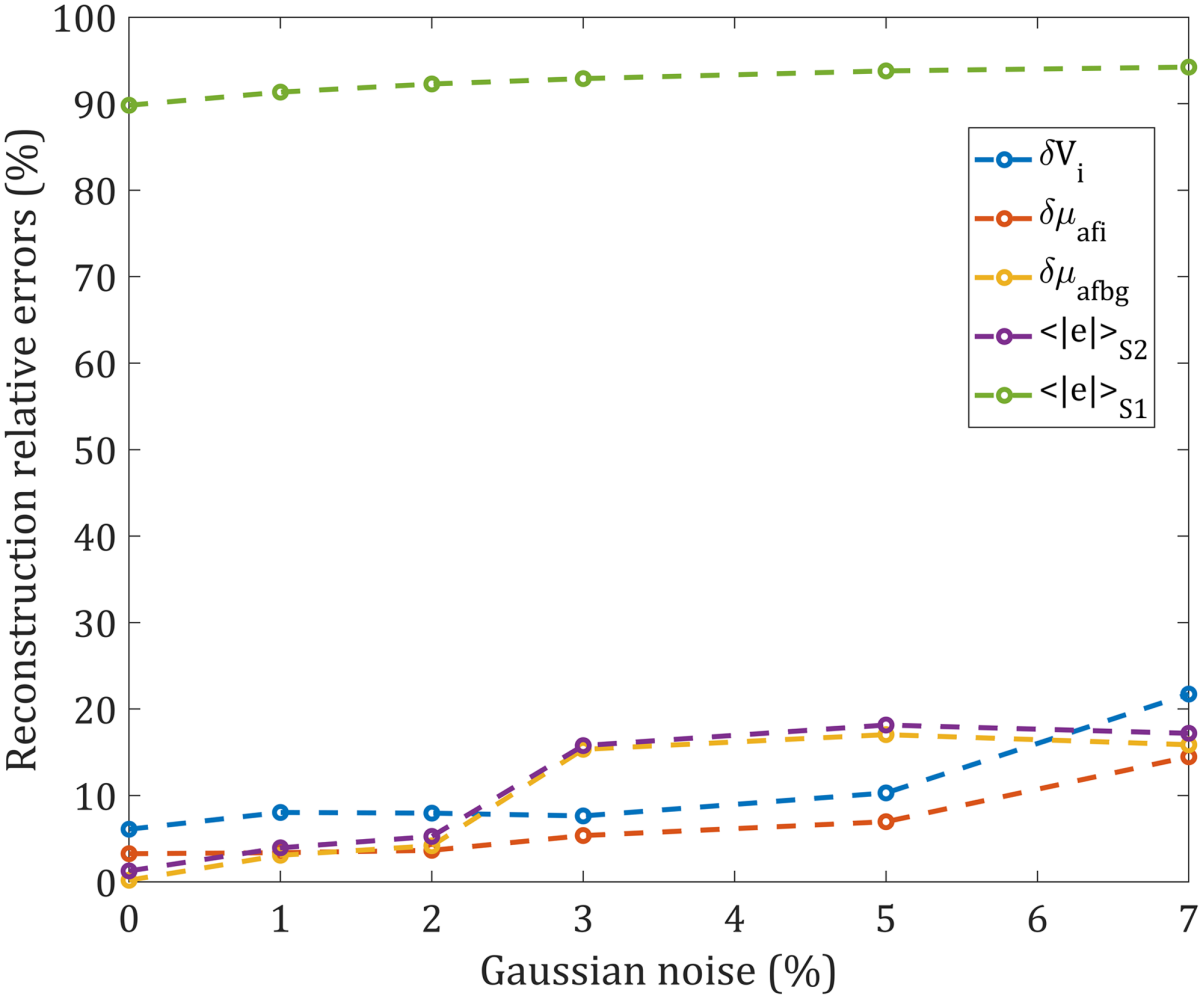
Reconstruction S2 relative errors ($\delta V_{i}$, $\delta \mu_{afi}$, $\delta \mu_{afbg}$, $\langle |e|\rangle_{S2}$) and S1 relative error ($\langle |e|\rangle_{S1}$) for different simulated noise levels (calculated at points where the Gaussian-profile noise amplitude factor was 0, 1%, 2%, 3%, 5%, and 7%). The same mesh was used for data generation and reconstruction (Mesh 1 from Table 1), whereas the matrix inversion was performed on a coarser 6480-node mesh.

Next, we incorporate noise in the analysis to see how the reconstruction performs in the face of uncertainty in input data. Noise in signals is a general term and can stem from the physical limitations of the detection system. The ratio of this noise compared with the useful signal can usually be reduced by increasing the excitation power or extending the measuring time. Other sources of noise come from our limited knowledge about the physical system, and from the model, which is likely very simplified compared with reality. Random variables representing the noise are added here to the theoretically computed signals (obtained by FEM). Every random variable (for every measurement point and both the excitation and the fluorescence emission wavelengths) is taken from a Gaussian distribution with a mean value of 0 and a standard deviation equal to the corresponding theoretically computed signal, multiplied with an amplitude factor, varied here from 0 to 7%. The Gaussian distribution for noise is chosen to model not only the statistics of photon detection (shot noise) but also any other uncertainties. For the comparison, in our CW detection system, a Gaussian noise with an amplitude factor of 1% corresponds to the Poisson noise for detected power of $4.5\cdot 10^{-10}\;W$, whereas 3% of amplitude factor for Gaussian noise corresponds to the detected power of $4.5\cdot 10^{-11}\;W$ for Poisson noise. The noise modeled by a Gaussian distribution with noise amplitude factors of 3% or more is a good way to model uncertainties even beyond the standard deviations of photon detection for relevant signal levels (see Sec. 4.2 for the infrared signal cut-off threshold). The reconstruction was performed on the same finite element mesh used for data generation to eliminate uncertainties due to FEM resolution limitations. Figure 6 was obtained by repeating the same method of generating noisy data 5× to find the averaged absolute values of the reconstruction relative errors.

The average relative errors for the reconstructed volume, fluorophore absorption coefficients of the inclusion, background, and every node in the mesh are plotted in Fig. 6. For the noise amplitude factor less or equal to 2%, all the relative errors from S2 reconstruction are less than 10% (all of them but volume with less than 6% relative error). It was observed that the reconstruction quality decreases when the noise amplitude factor is 3% or more. A noise amplitude of 10% already results in distorted reconstruction images, usually having more than the two expected regions, which have even higher errors (not shown in the graph, because the classification of the two expected regions is not relevant). It is possible to observe that the relative error of the volume reconstruction and the fluorophore absorption of the inclusion is still acceptable (<10%) as long as the noise amplitude factor is not >5%. The benefits of using S2 can be seen by comparing the average absolute values of the relative errors for S2 $\langle |e|\rangle_{S2}$, and S1 $\langle |e|\rangle_{S1}$, in Fig. 6. Although the S1 relative error is always around 90%, the S2 relative error is less than 6% for the noise amplitude factor less or equal to 2% or less than 20% for the noise amplitude factor between 3% and 7%.

For the parameters varied so far (fluorescent inclusion size, fluorophore concentration, and noise level), the S1 reconstruction average absolute value of the relative error was around 90%, and similar values have been reported in the literature.^24^^,^^52^ The necessity of using structural a priori information to obtain quantitatively accurate reconstructions was stated by Lin et al.^24^ Here, we demonstrated that it is possible to obtain quantitatively more accurate results if the second stage (see Sec. 2.5) is added to the reconstruction algorithm, even without any structural (geometrical) prior in the regularization (see Sec. 2.4). Similar approach to our proposed method, without any structural a priori information, was applied by Kwong et al.,^52^ using the technique called “temperature-modulated fluorescence tomography.” However, the important difference is that while there a functional a priori information was obtained by exploiting the thermal properties of specifically designed fluorophores, our proposed method uses only the results of S1 as an input for S2 and therefore more general fluorophores can be used. Both approaches resulted in comparable S2 reconstruction relative errors (up to around 15% for the fluorophore absorption coefficient and 35% for the volume).

Optical properties of the medium around the fibers are given as inputs to the reconstruction algorithm. If the forwarded (given) parameters do not match the actual optical properties of the medium, the reconstruction is affected. Table 4 shows to what extent. Although the absolute value of the relative error for the reconstructed volume is <26% in all but one of the cases considered, most of it naturally comes from the finite element mesh discretization of space, and the limited resolution for the volume calculation. More interesting is the error for the reconstructed fluorophore absorption coefficient of the inclusion. The fluorophore absorption is close to the GT value, within about 20%, for the optical properties closer to the default absorption of $0.50\;{\mathrm{cm}}^{-1}$ and reduced scattering of $8.7\;{\mathrm{cm}}^{-1}$ (the first seven rows of Table 4). Larger deviations, which can also occur in clinical reality, are reported in the last six rows of Table 4. The reconstructed fluorophore absorption coefficient is overestimated in the cases where the background absorption is overestimated or the background reduced scattering is underestimated, and vice versa.

**Table 4:** Simulated actual and given background absorption ($\mu_{a,\text{actual}}$, $\mu_{a,\text{given}}$), reduced scattering coefficients ($\mu_{s,\text{actual}}^{\prime }$, $\mu_{s,\text{given}}^{\prime }$), and the resulting relative errors of the reconstructed volume ($\delta V_{i}$) and fluorophore absorption coefficient of the inclusion ($\delta \mu_{\mathrm{afi}}$). The same Mesh 2 (Table 1) was used for data generation and reconstruction.

$\mu_{a,\text{actual}}$ (${\mathrm{cm}}^{-1}$)	$\mu_{a,\text{given}}$ (${\mathrm{cm}}^{-1}$)	$\mu_{s,\text{actual}}^{\prime }$ (${\mathrm{cm}}^{-1}$)	$\mu_{s,\text{given}}^{\prime }$ (${\mathrm{cm}}^{-1}$)	$\delta V_{i}$ (%)	$\delta \mu_{\mathrm{afi}}$ (%)
0.30	0.30	8.7	8.7	−12.0	4.7
0.30	0.50	8.7	8.7	−23.0	20.1
0.50	0.30	8.7	8.7	0.6	−10.2
0.50	0.50	8.7	8.7	−11.1	3.2
0.50	0.50	7.3	7.3	−11.7	3.8
0.50	0.50	7.3	8.7	−15.2	−6.8
0.50	0.50	8.7	7.3	−7.4	15.0
0.50	0.50	15.0	15.0	−5.5	0.3
0.50	0.50	15.0	8.7	−1.1	46.3
0.50	0.50	8.7	15.0	−18.0	−29.7
0.20	0.20	8.7	8.7	−12.8	5.7
0.20	0.50	8.7	8.7	−44.8	52.7
0.50	0.20	8.7	8.7	25.5	−21.5

**Fig. 7:**
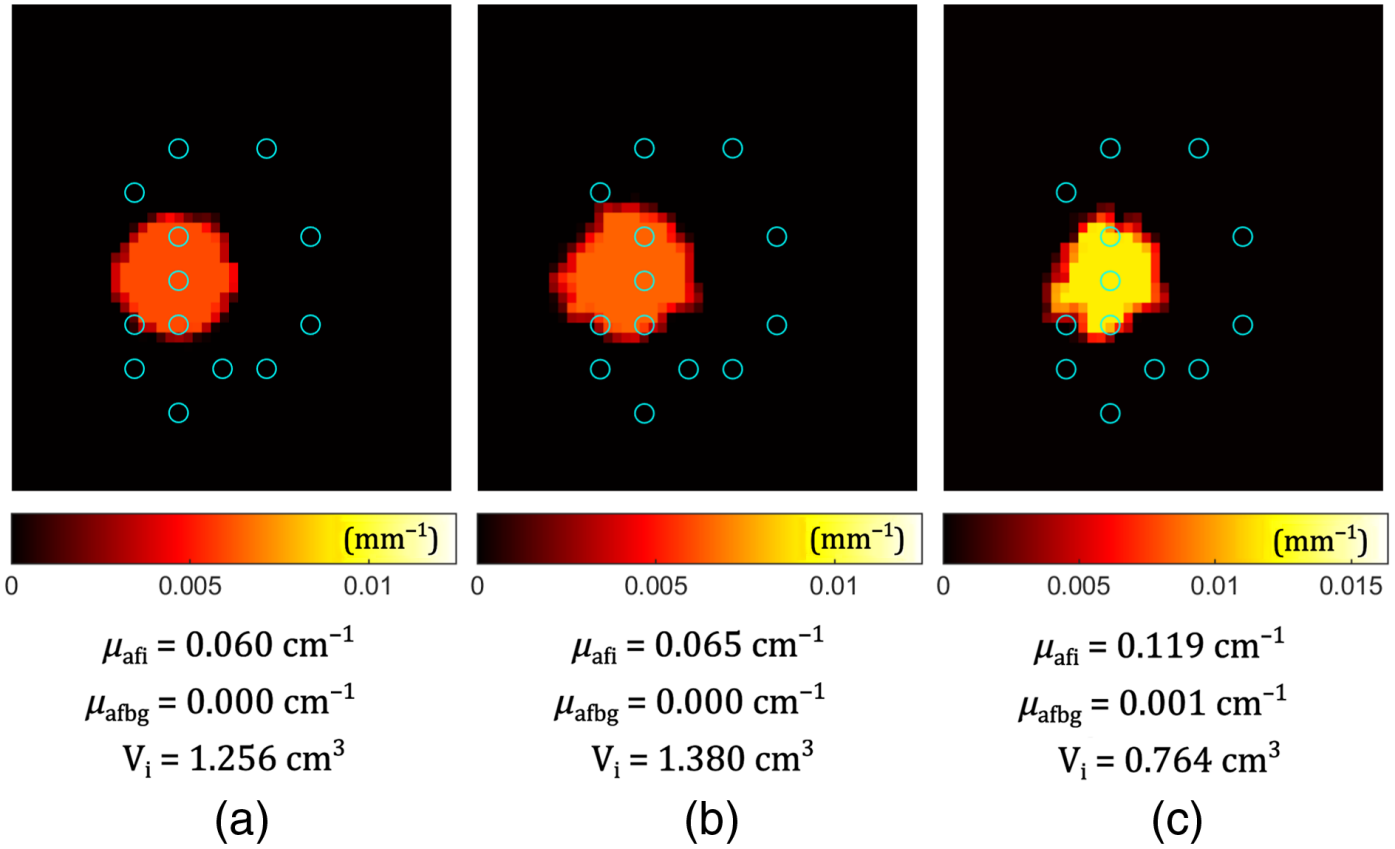
Simulation of the scenario where the absorption and the reduced scattering coefficients of the inclusion ($\mu_{a}$, $\mu_{s}^{\prime }$) differ from those of the background ($\mu_{ab}$, $\mu_{sb}^{\prime }$). Slice through the spherical inclusion center. Fluorophore absorption coefficients are shown with different colors. Blue circles represent fiber positions. Below the images, values are given for the fluorophore absorption of the inclusion $\mu_{\mathrm{afi}}$, fluorophore absorption of the background $\mu_{\mathrm{afbg}}$, and the volume of the inclusion $V_{i}$ for (a) GT case, (b) reconstruction with correct optical properties around the fibers, and (c) reconstruction with just homogeneous optical properties in the whole medium.

The fluorescent inclusions considered so far were different from the background only in terms of the fluorophore absorption coefficient, or the concentration of the fluorophores. Now we take into account the possibility that the inhomogeneity (spherical inclusion) added to the background may have different optical properties—absorption and scattering (inclusion: $\mu_{a}=0.20\;{\mathrm{cm}}^{-1}$ and $\mu_{s}^{\prime }=15.7\;{\mathrm{cm}}^{-1}$, background: $\mu_{ab}=0.50\;{\mathrm{cm}}^{-1}$ and $\mu_{sb}^{\prime }=7.3\;{\mathrm{cm}}^{-1}$). Figure 7 shows how the reconstruction is affected if the absorption and the reduced scattering coefficient of the medium are treated as homogeneous (c) in the whole medium (without specifying optical properties around each of the fibers), compared with the case where the correct input is given (after taking into account where the fibers are in respect to the spherical inclusion) (b). Note that this “correct” input is still imperfect in describing the medium optical properties. However, it is realistic because the IDOSE^8^^,^^18^ algorithm can estimate the properties around each fiber. The limiting factor is the mapping between the fiber coordinates and FEM node coordinates, i.e., mesh resolution. However, the reconstruction with more realistic optical properties is significantly better than that with plain, homogeneous optical properties (see Fig. 7). Similar conclusions about how the absorbing and scattering inhomogeneities affect the reconstructed fluorophore absorption have been reported by Abascal et al.^53^ and Soubret et al.^54^

Experimental data always contain a certain level of noise. For the measurement points with a low signal level (for example, when the detector fiber is far from the source fiber, or absorption in between the fibers is high), the signal-to-noise ratio can become so low that these measurement points become unreliable. It is possible that a fiber is partly blocked or acquires low signals. As a consequence, even though the fluorescent signal is low, the excitation wavelength signal is also low, and the Born ratio can become high because the “detected fluorescent signal” is dominated by background noise with a level comparable to or even a few orders of magnitude greater than the actual fluorescent signal. Such measurement points must not be forwarded to the reconstruction algorithm. Because the inverse problem is ill-posed, every unreliable data point can amplify the reconstruction error, resulting in a reconstructed image very different from the truth.

**Fig. 8:**
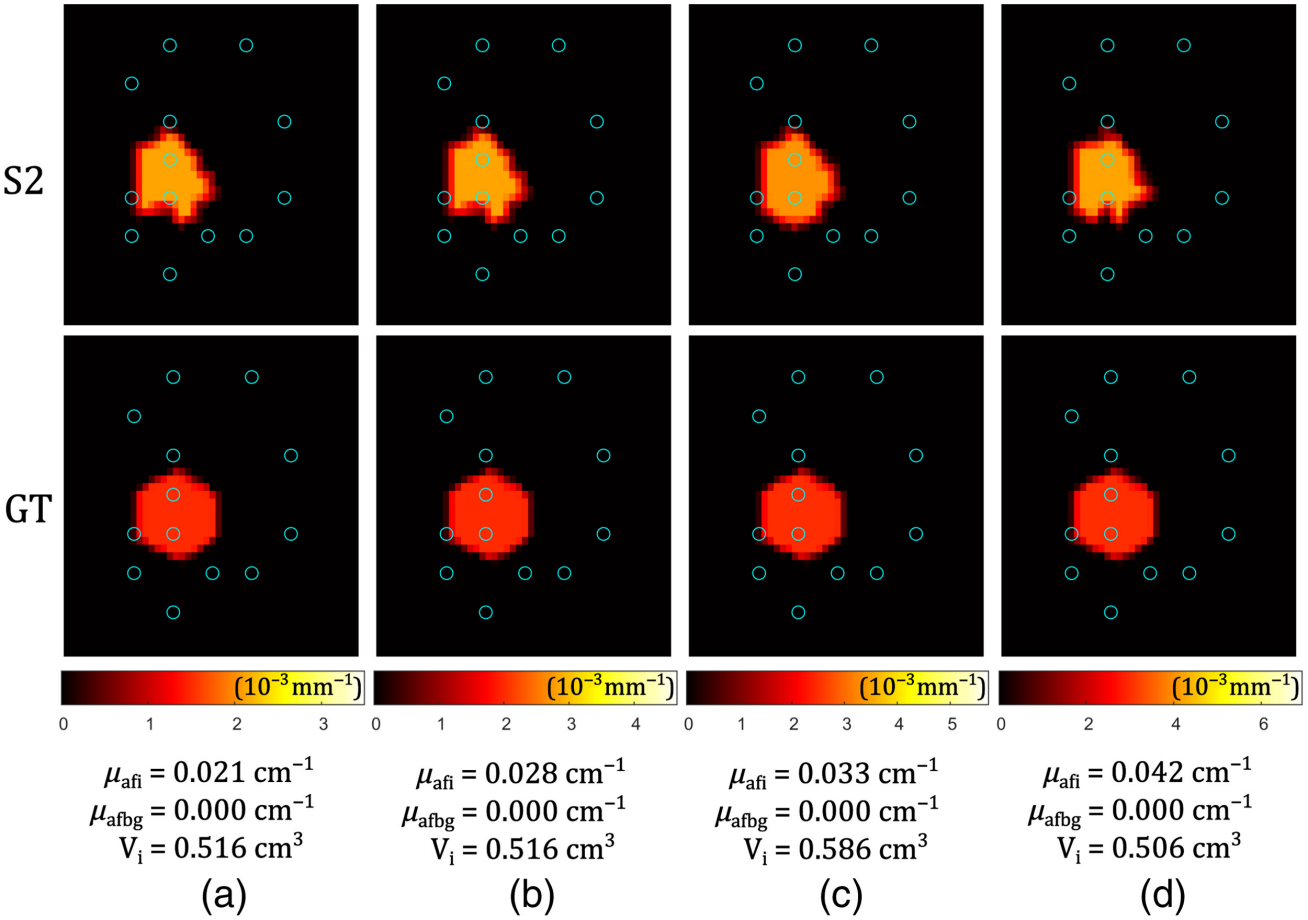
Simulation of instrument cut-off effect in a configuration with 13 fibers (Table 2). Blue circles represent fiber positions. Slice through the spherical inclusion center—comparison between the S2 reconstructed image and the GT. Below the images, reconstructed values are given for the fluorophore absorption of the inclusion $\mu_{afi}$, fluorophore absorption of the background $\mu_{afbg}$, and the volume of the inclusion $V_{i}$ for different GT fluorophore absorptions of the inclusion ($\mu_{afi,gt}$): (a) $0.015\;{\mathrm{cm}}^{-1}$, (b) $0.020\;{\mathrm{cm}}^{-1}$, (c) $0.025\;{\mathrm{cm}}^{-1}$, and (d) $0.030\;{\mathrm{cm}}^{-1}$. GT fluorophore absorption of the background was $\mu_{afbg,gt}=0$. Data were created using Mesh 3, and the reconstruction was performed using Mesh 4 from Table 1.

Here, we simulate the data processing of real measurements. All measured fluorescent signals below a certain threshold ($P_{\text{cut}}$) are discarded, leaving $N_{m}^{\prime }\leq N_{m}$ values. This means that the reduced measurement vector has $N_{m}^{\prime }$ rows, as well as the reduced Jacobian matrix ($N_{m}^{\prime }\times N_{n}$). Reconstruction was performed on four different scenarios—the same spherical inclusion size and position (R=5.5  mm, center at $(x_{C},y_{C},z_{C})=(20,22,18)\;\mathrm{mm}$), but different $\mu_{afi}$, equidistantly covering the interval from 0.015 to $0.030\;{\mathrm{cm}}^{-1}$. The optical properties of the inclusions were the same as for solid inclusions used in phantoms, and the optical properties of the background were the same as those used for liquid phantom preparation, see Sec. 3.5. The optical properties around the fibers were set accordingly. Reconstruction results are shown in Fig. 8. The GT volume is $V_{\mathrm{gt}}=0.697\;{\mathrm{cm}}^{3}$. It can be visually observed that in the xy-plane through the center of the sphere (z=18  mm), the fluorophore spatial extent is not fully reconstructed. This is a consequence of the data cut-off. The same is true in the perpendicular, z-direction. As a result, the whole volume of the inclusion is underestimated, leading to an overestimation of the fluorophore absorption coefficients. For the cases shown in Fig. 8, the reconstructed volume is (72 to 84)% of the GT volume, whereas the fluorophore absorption coefficient is overestimated by (33 to 42)%. The threshold for simulated measurement data cut-off was $P_{\text{cut}}=4.5\cdot 10^{-11}\;W$, resulting in $N_{m}^{\prime }=50$ (out of $N_{m}=156$) above-threshold measurement points in the case of the lowest concentration (a) and $N_{m}^{\prime }=61$ in the case of the highest concentration (d).

**Fig. 9:**
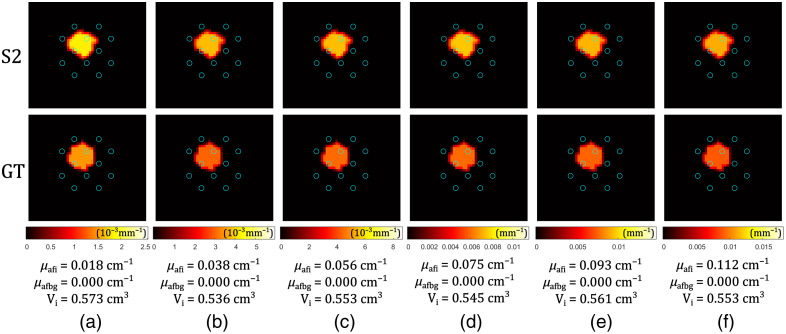
Simulation of instrument cut-off effect in a configuration with 12 fibers (Table 3). Blue circles represent fiber positions. Slice through the spherical inclusion center—comparison between the S2 reconstructed image and the GT. Below the images, reconstructed values are given for the fluorophore absorption of the inclusion $\mu_{afi}$, fluorophore absorption of the background $\mu_{afbg}$, and the volume of the inclusion $V_{i}$ for different GT fluorophore absorptions ($\mu_{afi,gt}$): (a) $0.015\;{\mathrm{cm}}^{-1}$, (b) $0.030\;{\mathrm{cm}}^{-1}$, (c) $0.045\;{\mathrm{cm}}^{-1}$, (d) $0.060\;{\mathrm{cm}}^{-1}$, (e) $0.075\;{\mathrm{cm}}^{-1}$, and (f) $0.090\;{\mathrm{cm}}^{-1}$. GT fluorophore absorption of the background was $\mu_{afbg,gt}=0$. Data were created using Mesh 5, and the reconstruction was performed using Mesh 6 from Table 1.

The effect of the instrument cut-off and the reconstructed volume underestimation described above can be diminished if the fibers are placed more favorably for solving the problem of DFT. The fiber configuration (see Table 2) considered in simulations is from a realistic clinical scenario. The priority in clinical PDT is to cause the photodynamic effect in tumorous tissue and only there. Neglecting this condition, we suggest another fiber configuration, with 12 fibers instead of 13, but having better spatial sensitivity around the spherical inclusion. The sphere’s center was at $(x_{C},y_{C},z_{C})=(22.5;27.5;20.0)\;\mathrm{mm}$, and the fiber coordinates are defined in Table 3. The same optical properties of the background and the inclusion, the same size of the inclusion, as used to obtain the results in Fig. 8, were applied in six reconstructions shown in Fig. 9. GT values for $\mu_{afi}$ varied from 0.015 to $0.090\;{\mathrm{cm}}^{-1}$ in equal steps. The cut-off threshold was kept $P_{\text{cut}}=4.5\cdot 10^{-11}\;W$, resulting in $N_{m}^{\prime }=54$ (out of $N_{m}=132$) above-threshold measurement points in the case of the lowest concentration (a), and $N_{m}^{\prime }=98$ in the case of the highest concentration (f). The reconstructed volume is (77 to 82)% of the GT volume, whereas the fluorophore absorption coefficient is overestimated by (21 to 27)%.

It is interesting to see how the reconstruction algorithm performs when the information on the actual fiber positions is uncertain. This uncertainty has one of the following two roots. First, in clinical practice, ultrasound imaging of needle and fiber tips has inherent measurement uncertainties, due to the needle bending or different lengths of fibers beyond the needle tips. Second, if the ultrasound is not used, as in our measurements on phantoms (see Sec. 4.2), there is no feedback on the actual fiber positions. The desired (aimed, planned) fiber coordinates are those chosen as optimal for the particular PDT treatment (see Sec. 3.2). The desired coordinates can be corrected by estimates of actual fiber positions based on ultrasound images, after fiber insertion into the patient’s body. These coordinates, denoted as $x_{i,\text{given}}$, $y_{i,\text{given}}$, and $z_{i,\text{given}}$, for i=1,…,13, and defined in Table 2, are then given as inputs to the tomographic reconstruction algorithm. On the other hand, $x_{i,\text{actual}}$, $y_{i,\text{actual}}$, and $z_{i,\text{actual}}$ are actual positions of inserted fibers, which can be more or less different from those given to the reconstruction algorithm. For this simulation, actual fiber coordinates were obtained according to: $x_{i,\text{actual}}=x_{i,\text{given}}+\delta x_{i}$, $y_{i,\text{actual}}=y_{i,\text{given}}+\delta y_{i}$, and $z_{i,\text{actual}}=z_{i,\text{given}}+\delta z_{i}$, where $\delta x_{i}$, $\delta y_{i}$ and $\delta z_{i}$ are random variables taken from uniform distributions on intervals defined in Table 5. In this simulation, two realistic levels of error amplitudes were considered, each with seven random realizations (scenarios) of actual fiber positions. Table 5 shows the average relative errors for the reconstructed volume and fluorophore absorption coefficients of the inclusion and the background. Bigger coordinate uncertainties lead to larger reconstruction errors, with some scenarios resulting in more than two regions. It can be concluded that the reconstruction is sensitive to the correct knowledge of the fiber positions, and to have accurate reconstruction, only limited errors in the fiber position estimation can be accepted.

**Table 5:** Simulated errors ($\delta x_{i}$, $\delta y_{i}$, $\delta z_{i}$) of the estimation of actual fiber coordinates, random variables uniformly distributed over the defined intervals, with the resulting average relative errors for the reconstructed volume ($\delta V_{i}$) and fluorophore absorption coefficients of the inclusion ($\delta \mu_{\mathrm{afi}}$) and the background ($\delta \mu_{\mathrm{afbg}}$), for seven scenarios (averaged over the number of scenarios resulting in the specific number of regions). The same Mesh 2 (Table 1) was used for data generation and reconstruction.

$\delta x_{i}$ (mm)	$\delta y_{i}$ (mm)	$\delta z_{i}$ (mm)	Regions	Scenarios	$\delta V_{i}$ (%)	$\delta \mu_{\mathrm{afi}}$ (%)	$\delta \mu_{\mathrm{afbg}}$ (%)
[−0.5;0.5]	[−0.5;0.5]	[−1;1]	2	5	−8.8	5.5	2.0
[−0.5;0.5]	[−0.5;0.5]	[−1;1]	3	2	6.2	−6.2	−16.0
[−1;1]	[−1;1]	[−2;2]	2	5	−23.6	16.8	3.6
[−1;1]	[−1;1]	[−2;2]	3	2	−41.9	33.4	−15.0

**Fig. 10:**
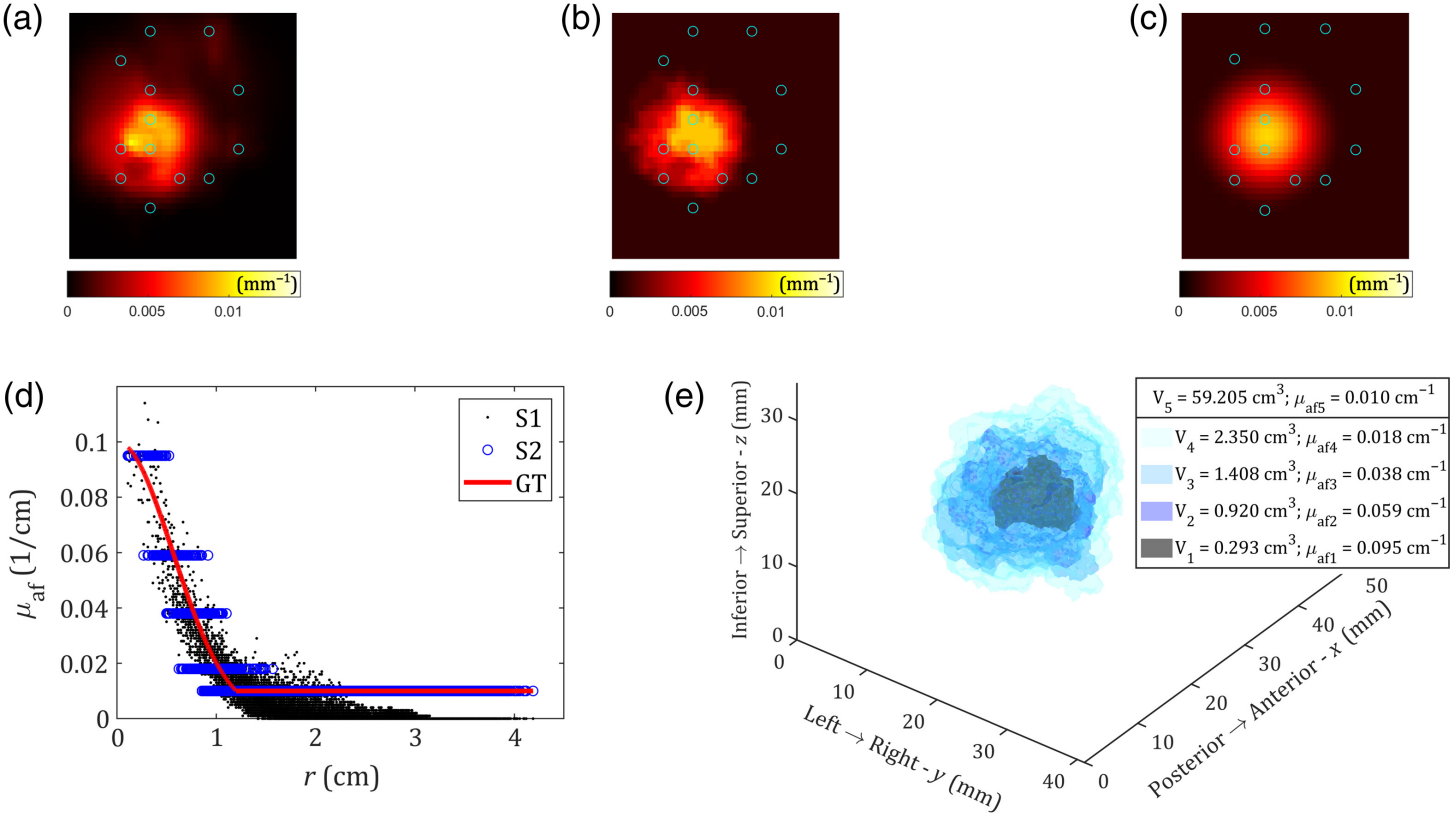
Simulation of the scenario where the fluorophore absorption coefficient of the spherical (R=12  mm) inclusion follows a Gaussian profile, with the peak $\mu_{\mathrm{afi}}=0.10\;{\mathrm{cm}}^{-1}$ at the sphere center $(x_{C},y_{C},z_{C})=(20,22,18)\;\mathrm{mm}$ and background outside of the sphere $\mu_{\mathrm{afbg}}=0.01\;{\mathrm{cm}}^{-1}$. The plane cuts perpendicular to the z-axis, at z=18  mm, are shown as color maps for (a) S1 reconstruction results, (b) S2 reconstruction results, and (c) GT. Blue circles represent fiber positions. (d) $\mu_{af}(r)$ dependence calculated at mesh nodes, where r is the distance of the node from the sphere center, for S1 reconstruction (black dots), S2 reconstruction (blue circles), and GT Gaussian $\mu_{af}(r)=\mu_{\mathrm{afi}}\;\exp(-\ln(\mu_{\mathrm{afi}}/\mu_{\mathrm{afbg}})r^{2}/R^{2})$ (red solid line). (e) 3D representation of S2 results. Data were generated using the denser Mesh 3, and the inverse problem was solved using Mesh 4 (see Table 1), whereas the matrix inversion was performed on a coarser 7140-node mesh.

Finally, instead of a homogeneous fluorophore concentration inside the sphere and in the background, with a step-like transition between the two, we could consider a continuous transition from the highest concentration at the sphere center, to the constant background level concentration outside of the sphere of radius R=12  mm. The continuous transition has the Gaussian profile [solid line in Fig. 10(d)]. From Fig. 10(d), it can be seen that the background fluorophore absorption is accurately reconstructed in S2, which is an improvement compared with S1. Although it is evident that a few homogeneous regions are not enough to accurately approximate the Gaussian profile, reconstructed discrete values for $\mu_{af}$ are not far from GT values. The number and size of regions depend on adaptive thresholds. Specifically in this simulation, two degrees of freedom for the peak ($t_{p}$) and the background threshold ($t_{b}$) were allowed, i.e., $t_{p}$ and $t_{b}$ can have mutually different values under the condition that $t_{b}\leq t_{p}$, and both $t_{p}$ and $t_{b}$ are from the defined set of discrete values (see Sec. 3.1). Figure 10(e) shows reconstructed regions with their volumes and fluorophore absorption coefficients in 3D.

### 4.2 Phantom Validation

The optical and geometrical properties of the realized phantom are described in Sec. 3.5. The gelatin-based spherical inclusions of seven different verteporfin concentrations were tested: $c_{0}=0$, $c_{1}=0.3\;\mathrm{mg}/\mathrm{kg}$, $c_{2}=0.6\;\mathrm{mg}/\mathrm{kg}$, $c_{3}=0.9\;\mathrm{mg}/\mathrm{kg}$, $c_{4}=1.2\;\mathrm{mg}/\mathrm{kg}$, $c_{5}=1.5\;\mathrm{mg}/\mathrm{kg}$, and $c_{6}=1.8\;\mathrm{mg}/\mathrm{kg}$. The experimental procedure is described in Sec. 3.6. The fiber configuration used is defined in Table 2. The center of the spherical inclusion was placed at $(x_{C},y_{C},z_{C})=(22.5,27.5,20.0)\;\mathrm{mm}$. For each spherical inclusion, the measurement had five sequences, and the average was taken to increase the signal-to-noise ratio. Before forwarding the data to the reconstruction algorithm, raw measurement data were processed such that every infrared detected value below $4.5\cdot 10^{-11}\;W$ was discarded, resulting in the reduced vector of measurements having $N_{m}^{\prime }=46$ elements (out of $N_{m}=132$) in the case of concentration $c_{1}$, and $N_{m}^{\prime }=75$ in the case of concentration $c_{6}$. This cutting-off is done to make the reconstruction more robust by avoiding amplification of measurement points with low levels of detected infrared power, as explained in Sec. 4.1.

**Fig. 11:**
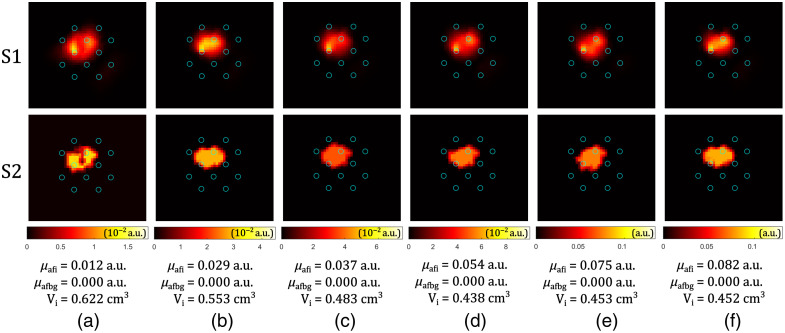
Tomographic reconstruction of the fluorophore absorption, cut plane z=20  mm, for different verteporfin concentrations applied in the gelatin spheres (c): (a) 0.3  mg/kg, (b) 0.6  mg/kg, (c) 0.9  mg/kg, (d) 1.2  mg/kg, (e) 1.5  mg/kg, and (f) 1.8  mg/kg. Results of the two stages of the reconstruction are shown (S1 and S2) in different rows, the values for the reconstructed fluorophore absorption coefficient of the inclusion $\mu_{\mathrm{afi}}$, background $\mu_{\mathrm{afbg}}$, and the estimated volume of the inclusion $V_{i}$ are given below. Blue circles represent fiber positions. Columns (a)–(f) have different color bars.

The tomographic reconstruction of fluorophore absorption was performed on Mesh 6 from Table 1, and a lower resolution 6137-node mesh was used for matrix inversion. Reconstruction results for six nonzero verteporfin concentrations are shown in Fig. 11, with the plane cut through the sphere center (z=20  mm). The reconstructed volume of the inclusion is underestimated as expected, (63 to 89)% of the ideal sphere volume (Sec. 4.1). The reconstructed fluorophore absorption coefficient is expressed in arbitrary units for two reasons. First, the reconstructed volume is not reliable, and when the volume is underestimated, the fluorophore absorption coefficient is overestimated. However, all the reconstructed volumes are close to each other, and the arbitrary unit can be used for relative comparison of the reconstructed PS drug concentrations. Second, the fluorescence quantum yield, defined in Sec. 2.1, was assumed to be γ=10%.^55^^,^^56^ If this value is wrong, all the results obtained from experimental measurements cannot be expressed in a specific SI unit, but in a scaled unit which can be used for a relative comparison.

**Fig. 12:**
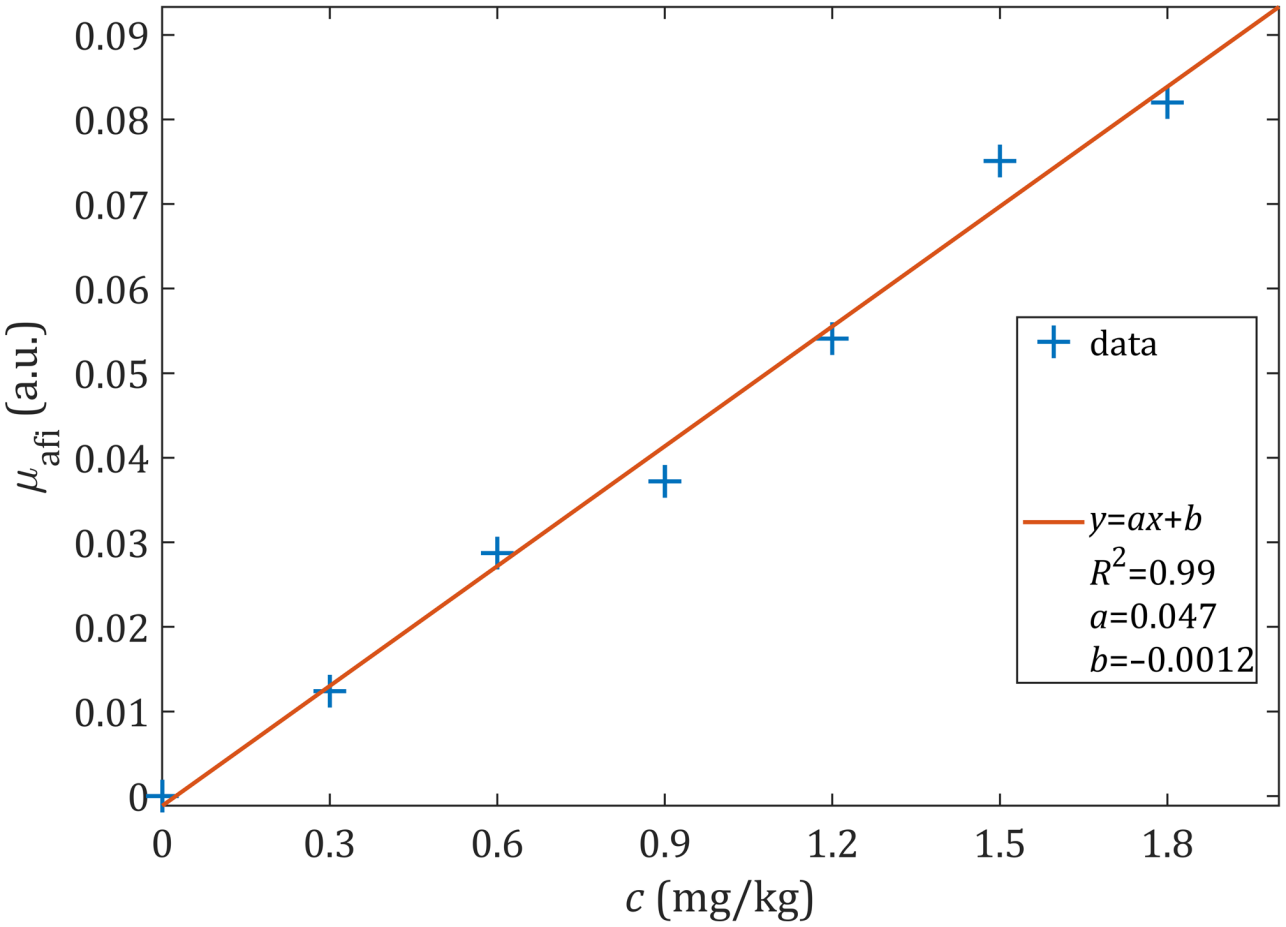
Reconstructed fluorophore absorption coefficient ($\mu_{afi}$) of solid spherical inclusions for different applied concentrations of verteporfin (c). Experimental data marked with +, parameters of the linear fit (red line) shown in the legend.

The relative comparison of the reconstructed fluorophore absorption coefficients for seven verteporfin concentrations (including zero) applied in phantoms is graphically shown in Fig. 12. A linear (y=ax+b) least-squares fitting was performed through seven data points, resulting in the coefficient of determination $R^{2}=0.99$, and the free term b=−0.0012, suggesting a direct proportionality between the fluorophore absorption and the fluorophore concentration. The obtained linearity parameters are comparable with those from other fluorescence tomography quantification methods.^57^^,^^58^ Our tomographic reconstruction algorithm validated on verteporfin phantoms shows the potential for capturing quantitative differences in the PS drug concentration by using SpectraCure’s P18 clinical system.

### 4.3 Limitations and Potential Improvements

The main limitations of the suggested DFT reconstruction methods are determined by the mathematical nature of the problem and the physical limitations of the measurement process. There is also a computational factor that plays an important role in potential real-time clinical applications. To have a better spatial resolution, more nodes should be added to the finite element mesh, and this requires more measurement points, i.e., source-detector pairs. Physically, because the prostate volume is already small and the tumor region is even smaller, placing many brachytherapy needles of ∼1  mm diameter is not a simple task for a clinician. Moreover, the high spatial density of metal needles inside a relatively small tissue volume affects the light propagation and the model derived from the diffusion equation. SpectraCure’s P18 system used in experiments has 18 photonics modules; therefore, slots for up to 18 optical fibers, which is more than enough for most prostate cancer PDT applications, but imposes a theoretical limit of a maximum 18·17=306 measurement points for DFT.

The time required to solve the inverse problem in two stages, having as inputs the measurements expressed as Born ratios and the optical properties estimated around the fibers, is around 4 to 5 min on CPU Intel i9, 10th generation, 3.7 GHz, with 32 GB RAM. The computations can be faster or slower, depending on the finite element mesh resolution. The choice of the finite element mesh is important and it has to take into account the dimensions of the medium, the number of fibers, and time constraints (in the case of real-time clinical applications). For a clinically realistic measurement with a set of 12 to 13 fibers interstitially placed inside a volume of around $(5\times 5\times 5)\;{\mathrm{cm}}^{3}$, finite element meshes of around 20,000 to 30,000 nodes were used.

Data acquisition by this system can still be improved. Increasing the numerical aperture of the fibers is desired, and already in the latest system, NA=0.37 is used, which is better than NA=0.22 used in this work (see Sec. 3.6). Leakage of 690 nm light through filters to the infrared detectors should be rejected as much as possible or precisely estimated and compensated for. To make the reconstruction algorithm work robustly, regardless of noise and expected errors in detection, a certain threshold below which all the infrared signals are to be discarded, should be carefully determined. It would be useful to predict in simulations how much the reconstructed volume is underestimated, as a consequence of this cutting-off, and take it into account as a correction for the reconstruction from real measurement data.

Optical properties around the fibers are estimated according to already developed methods.^8^^,^^18^ This is a good input to the fluorescence reconstruction algorithm but could be improved in terms of spatial resolution and decoupling of absorption from the scattering. However, that would result in more complex problems and a potential need for time-domain systems. On the other hand, if it is of interest to reduce the computational complexity and achieve faster reconstructions, simpler models can be developed by assuming a homogeneous background medium, and analytical expressions are given in Sec. 2.1, removing the need for FEM calculations.

Note that in the current model, the effect of metal needles and the optical properties of silica fibers is not considered. We believe that this will not affect the reconstruction significantly, because the effective optical properties around the fibers are estimated as mentioned above and the Born ratio is used to express the measured fluorescence. Light propagation at fluorescent wavelengths is not expected to differ significantly from light propagation at the excitation wavelength. As the Born ratio of the detected fluorescent and excitation wavelength signals is forwarded to the reconstruction algorithm, all uncertainties including the inhomogeneous or different optical properties in the medium and in small volumes occupied by the fibers and needles, are expected to be canceled or negligible. However, it is suggested to model the presence of optical fibers and brachytherapy needles in the medium, but the diffusion equation approach would become more complex. Another possible approach is to compare the effects on the Born ratio in Monte Carlo simulations in the two cases—when the fibers and needles are modeled and when they are not.

A precise knowledge of the fiber positions is important, as it is an input to the forward model used in reconstructions. The feedback information about the fiber positions is available in the clinical setting, obtained from the ultrasound probe. This was not available in phantom experiments, and we used recommended (aimed, desired) fiber positions as the actual fiber positions (see Secs. 3.2 and 4.1). In the reality of the experiments with phantoms, because the gelatin sphere was attached to the fibers, precise positioning of all the fibers and the sphere relative to them was not possible. More precise information about the actual fiber positions would improve the accuracy of the forward model, which is expected to result in better reconstruction of the PS drug concentration.

Moreover, it would be beneficial to separately characterize fluorophore inclusions, before the tomographic reconstruction in a liquid background, to verify that the concentration of fluorophores (PS drug) is uniformly distributed over the inclusion volume. The phantom used in this paper had a simplified geometry, unlike the hybrid phantom from our previous work^26^ whose prostate boundaries were defined using the 3D model obtained from the clinical ultrasound data. The effect of such geometry could be explored and considered in the future.

Finally, the algorithm for determining the regions as inputs to S2 could be implemented in many different ways. One direction for further exploration is to extend the allowed set for adaptive threshold fractions $t_{p}$ and $t_{b}$, for example by not imposing $t_{p}=t_{b}$, including more than nine different values, or extending the empirically determined interval (0.33, 0.87) (Sec. 3.1). Other suggested directions include application of clustering algorithms based on statistical pattern recognition or artificial neural networks. Moreover, it is expected that the structural a priori information in S1 (which can be obtained from ultrasound during the treatment) would improve the accuracy of the inputs for S2 and therefore the final results of S2.

## 5 Conclusion

We have proposed a novel two-stage approach for estimating the spatial distribution of the fluorescent PS drug starting from CW measurement data at excitation and fluorescence emission wavelengths, acquired by interstitially placed optical fibers. S1 of the proposed algorithm relies on standard DOT reconstruction methods, without any geometrical prior. The results from S1 are then used as inputs for S2, presented here for the first time. The reconstruction methods were implemented in MATLAB, using the NIRFAST package. Numerical simulations in various scenarios were performed to test the newly developed methods and find their limitations. As found in simulations with a single homogeneous fluorescent spherical inclusion, it is essential to increase the signal-to-noise ratio to have a reliable reconstruction of the volume of the inclusion and its fluorophore absorption coefficient. Various inclusion sizes and fluorophore concentrations were investigated. More accurate results are obtained if the optical properties around the fibers are correctly taken into account, compared with the case where a mismatch between the actual optical properties and those forwarded to the reconstruction algorithm exists. The novel two-stage approach resulted in the average absolute value of the reconstruction relative error of the order of (1 to 10)%, compared with around 90% in a standard single-stage approach. The tomographic reconstruction methods were validated on prostate tissue-mimicking phantoms to estimate the spatial distribution and concentration of fluorescent PS verteporfin used in the PDT of prostate cancer. The reconstructed fluorophore absorption coefficients had a good direct proportionality with the applied concentrations of verteporfin, whereas the reconstructed volume of the inclusion had a relative error (10 to 35)%. The results from experimental measurements show the potential for further development of the proposed DFT methods and their eventual clinical in vivo application for monitoring the PS drug concentration in real-time during the PDT of tumors.

Before transferring to clinics, these methods should be validated on the next generations of phantoms of different shapes, sizes, PS concentrations, and with various fiber configurations. In parallel, the reconstruction methods can be used offline to find the correlation between the prostate cancer treatment outcome and the amount of the PS and its spatial and temporal evolution in already finished treatments.

## Data Availability

The data presented in this article are publicly available in Zenodo at https://doi.org/10.5281/zenodo.14713507. The software code may be available upon a reasonable request to the corresponding author.
